# Epigenomic mapping reveals distinct B cell acute lymphoblastic leukemia chromatin architectures and regulators

**DOI:** 10.1016/j.xgen.2023.100442

**Published:** 2023-11-20

**Authors:** Kelly R. Barnett, Robert J. Mobley, Jonathan D. Diedrich, Brennan P. Bergeron, Kashi Raj Bhattarai, Alexander C. Monovich, Shilpa Narina, Wenjian Yang, Kristine R. Crews, Christopher S. Manring, Elias Jabbour, Elisabeth Paietta, Mark R. Litzow, Steven M. Kornblau, Wendy Stock, Hiroto Inaba, Sima Jeha, Ching-Hon Pui, Charles G. Mullighan, Mary V. Relling, Shondra M. Pruett-Miller, Russell J.H. Ryan, Jun J. Yang, William E. Evans, Daniel Savic

**Affiliations:** 1 Hematological Malignancies Program, St. Jude Children’s Research Hospital, Memphis, TN 38105, USA; 2 Department of Pharmacy and Pharmaceutical Sciences, St. Jude Children’s Research Hospital, Memphis, TN 38105, USA; 3 Graduate School of Biomedical Sciences, St. Jude Children’s Research Hospital, Memphis, TN 38105, USA; 4 Department of Pathology, University of Michigan–Ann Arbor, Rogel Cancer Center, Ann Arbor, MI 48109, USA; 5 Center for Advanced Genome Engineering, St. Jude Children’s Research Hospital, Memphis, TN 38105, USA; 6 Alliance Hematologic Malignancy Biorepository, Clara D. Bloomfield Center for Leukemia Outcomes Research, Columbus, OH 43210, USA; 7 Department of Leukemia, The University of Texas M. D. Anderson Cancer Center, Houston, TX, USA; 8 Department of Oncology, Montefiore Medical Center, Bronx, NY 10467, USA; 9 Division of Hematology, Department of Medicine, Mayo Clinic, Rochester, MN 55905, USA; 10 University of Chicago Comprehensive Cancer Center, Chicago, IL 60637, USA; 11 Department of Oncology, St. Jude Children’s Research Hospital, Memphis, TN 38105, USA; 12 Department of Pathology, St. Jude Children’s Research Hospital, Memphis, TN 38105, USA; 13 Department of Cell and Molecular Biology, St. Jude Children’s Research Hospital, Memphis, TN 38105, USA; 14 Integrated Biomedical Sciences Program, University of Tennessee Health Science Center, Memphis, TN 38105, USA

**Keywords:** acute lymphoblastic leukemia, ATAC-seq, chromatin accessibility, gene regulation, gene-regulatory network, transcription factor, transcription factor footprints, genetic variation, ATAC-QTLs

## Abstract

B cell lineage acute lymphoblastic leukemia (B-ALL) is composed of diverse molecular subtypes, and while transcriptional and DNA methylation profiling has been extensively examined, the chromatin landscape is not well characterized for many subtypes. We therefore mapped chromatin accessibility using ATAC-seq in primary B-ALL cells from 156 patients spanning ten molecular subtypes and present this dataset as a resource. Differential chromatin accessibility and transcription factor (TF) footprint profiling were employed and identified B-ALL cell of origin, TF-target gene interactions enriched in B-ALL, and key TFs associated with accessible chromatin sites preferentially active in B-ALL. We further identified over 20% of accessible chromatin sites exhibiting strong subtype enrichment and candidate TFs that maintain subtype-specific chromatin architectures. Over 9,000 genetic variants were uncovered, contributing to variability in chromatin accessibility among patient samples. Our data suggest that distinct chromatin architectures are driven by diverse TFs and inherited genetic variants that promote unique gene-regulatory networks.

## Highlights

• Identification of accessible chromatin in primary B-ALL cells from 156 patients
• TF-target gene interactions enriched in B-ALL are linked to cell proliferation
• Distinct TF footprint profiles are associated with subtype-specific chromatin architecture
• Thousands of inherited genetic variants identified impacting chromatin state

## Graphical abstract

**Figure:**
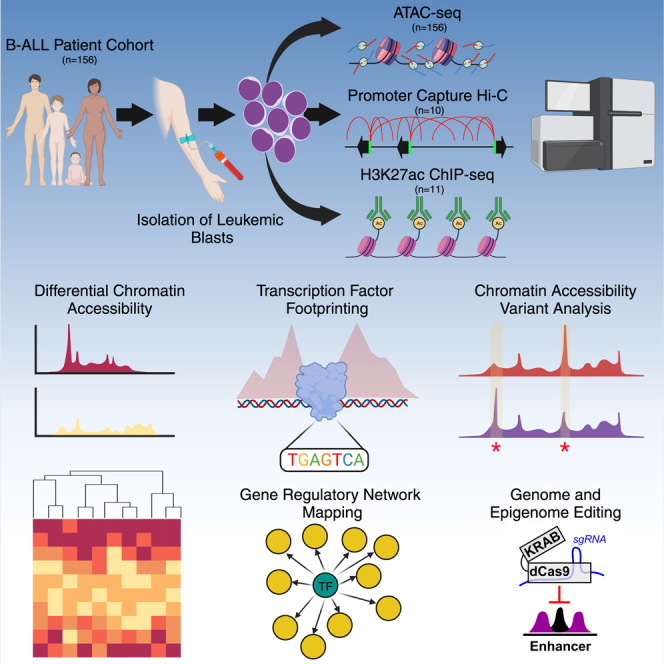

## Teaser

Barnett et al. profile the chromatin-accessibility landscape across B cell lineage acute lymphoblastic leukemia (B-ALL) patient samples, identifying differences among individual patients, B-ALL subtypes, and normal B cell progenitors. Utilization of transcription factor (TF) footprint profiling and promoter capture Hi-C yielded TF-binding patterns and TF-gene associations that define B-ALL.

## Introduction

Acute lymphoblastic leukemia (ALL) is derived from B and T cell lineage precursor cells and is the most common childhood cancer.^1^ A majority of acute lymphoblastic leukemias are derived from B cell lineages (B-ALL) that are comprised of distinct molecular subtypes characterized by unique chromosomal lesions, including aneuploidy, translocations, gene fusions, point mutations, and other chromosomal rearrangements that drive leukemogenesis.^2^ Numerous studies have identified extensive heterogeneity in transcriptomes^3^^,^^4^ and DNA methylomes^5^^,^^6^ among B-ALL subtypes in large patient cohorts, but there is limited understanding of chromatin landscapes. Here we provide an extensive survey of accessible chromatin state and *cis*-regulatory element activity in primary B-ALL cells from more than 150 patients across the United States.

Chromatin accessibility or open chromatin is a hallmark of active *cis*-regulatory elements that control spatial and temporal gene expression.^7^ Because ALL typically involves mutations (*PAX5-*altered), complex rearrangements (e.g., *DUX4-*rearranged, *PAX5-*altered, *ZNF384-*rearranged), and/or oncogenic gene fusions (e.g., *ETV6*::*RUNX1*, *TCF3*::*PBX1*, *KMT2A-*rearranged) of transcription factor (TF) genes, as well as disruptions of *cis*-regulatory elements,^8^ chromatin-accessibility maps can provide valuable information to better understand the leukemogenic process. Accessible chromatin sites can be mapped using transposases by performing assays for transposase-accessible chromatin with high-throughput sequencing (ATAC-seq).^9^^,^^10^ Although DNase treatment has also been used,^11^ one key advantage of ATAC-seq is the low sample input requirements compared to DNase-based assays. This makes ATAC-seq an attractive assay for mapping open chromatin in primary cells from patients wherein sample availability is limited. Additionally, chromatin accessibility allows for identification of bound TFs through an examination of TF footprints, which are defined by depletion in DNA transposition^12^ or DNase^13^ cleavage events within regions of accessible chromatin signal. As a result, the underlying TF-binding gene-regulatory networks that promote chromatin accessibility and differential gene expression can be predicted.

Previous large-scale studies of chromatin accessibility in primary cells have predominantly focused on distinct cell types^10^^,^^14^ or distinct tumor types and locations.^15^^,^^16^ Therefore, large-scale analyses aimed at better understanding the chromatin state in a single heterogeneous malignancy are currently lacking. To address this knowledge gap, we mapped chromatin accessibility in fresh primary ALL cells from 156 patients across ten molecular subtypes of B-ALL (*BCR*::*ABL1*, *DUX4-*rearranged, *ETV6*::*RUNX1*, high hyperdiploid, low hypodiploid, *KMT2A-*rearranged, *BCR*::*ABL1*-like [Ph-like], *PAX5-*altered, *TCF3*::*PBX1*, and *ZNF384-*rearranged) and B-other patient samples. Notably, these subtypes span the entire spectrum of clinical prognoses, including patients with excellent (*DUX4-*rearranged, *ETV6*::*RUNX1*, high hyperdiploid), good (*TCF3*::*PBX1*), intermediate (*ZNF384-*rearranged, *PAX5-*altered), and poor (*BCR*::*ABL1*, low hypodiploid, *KMT2A-*rearranged, Ph-like) prognosis. We also mapped histone H3 lysine 27 acetylation (H3K27ac) enrichment using chromatin immunoprecipitation sequencing (ChIP-seq) and performed promoter capture Hi-C in a subset of these patient samples to additionally infer functional activity and candidate target genes of accessible chromatin sites.

Using ATAC-seq chromatin-accessibility and histone profiling in primary ALL cells, we mapped *cis*-regulatory element activity in B-ALL. In complement to chromatin-accessibility profiling, we identified thousands of chromatin loops targeting promoters in multiple B-ALL cell lines to better inform linkages of *cis*-regulatory elements to cognate genes. We coupled these maps to TF footprints at accessible chromatin sites to identify key TFs and gene-regulatory networks across B-ALL samples and within distinct B-ALL subtypes. Our results identified extensive chromatin reprogramming between B cell progenitors and B-ALL as well as extensive heterogeneity in accessible chromatin landscapes among B-ALL subtypes. Specifically, we uncovered a focused subset of over 42,000 B-ALL open chromatin sites exhibiting extensive subtype enrichment and subtype-enriched TF-binding events. Notably, these sites can predict and classify B-ALL samples with 86% cross-validation accuracy. We additionally explored the impact of inherited genetic variation on the chromatin state and delineated over 9,000 ATAC-seq chromatin-accessibility quantitative trait loci (ATAC-QTLs) in B-ALL cells, including a subset that alters neighboring gene expression. Using this expansive B-ALL chromatin-accessibility dataset, our data collectively support substantial subtype specificity in chromatin accessibility that is driven in part by distinct TFs, as well as pronounced inter-individual heterogeneity in chromatin state through inherited genetic variants. Our work further supports the role of these distinct chromatin architectures in establishing unique gene-regulatory networks that impact gene expression and B-ALL cell biology.

## Results

### Chromatin-accessibility profiles of B-ALL patient samples spanning multiple subtypes

**Figure 1:**
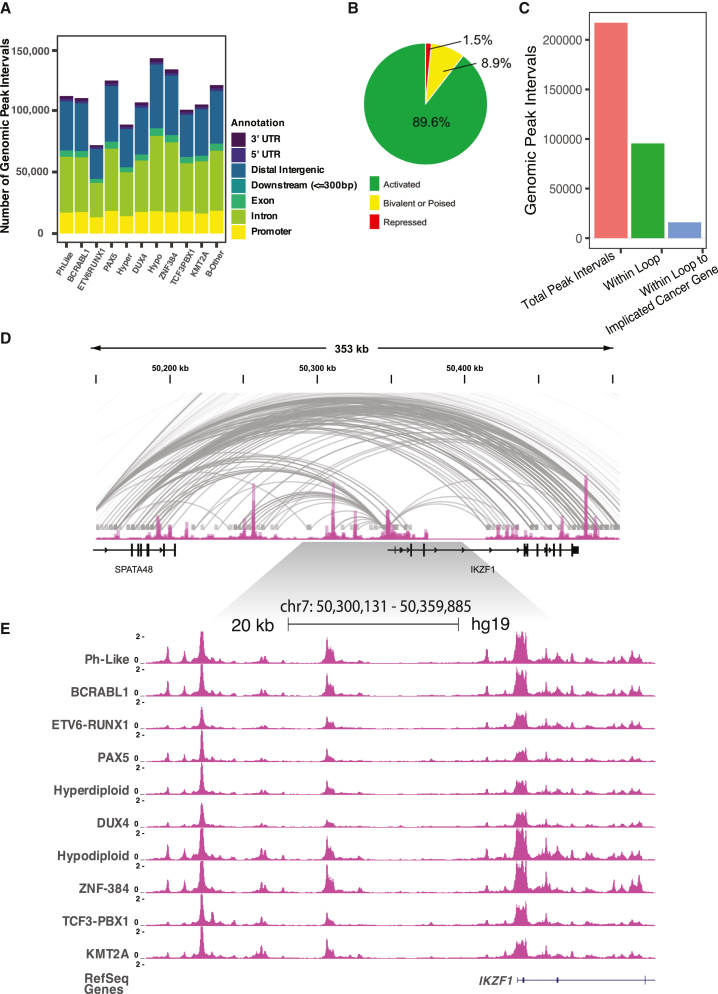
Chromatin-accessibility landscapes in B-ALL (A) Number and genomic location of accessible chromatin sites for ten B-ALL subtypes and B-other samples is provided. (B) Percentage of B-ALL accessible chromatin sties that map to H3K4me1 and/or H3K27ac active histone marks (active; green), H3K27me3 and H3K4me1 and/or H3K27ac bivalent or poised histone marks (bivalent or poised; yellow), and H3K27me3 only repressed histone marks (repressed; red). (C) B-ALL cell line chromatin loops detected using promoter capture Hi-C at B-ALL accessible chromatin sites. The total number of B-ALL accessible chromatin sites, number of B-ALL accessible chromatin sites within loops, and total number of accessible chromatin sites with a loop to a gene implicated in cancer is shown. (D) University of California Santa Cruz (UCSC) genome browser ATAC-seq signal track of average B-ALL chromatin accessibility and promoter capture Hi-C loops across the *IKZF1* gene locus. (E) UCSC genome browser ATAC-seq signal tracks of ten merged B-ALL subtypes with known molecular drivers across the *IKZF1* gene locus.

ATAC-seq using the Fast-ATAC^10^ method was performed on recently harvested primary ALL cells from 156 patients spanning ten B-ALL molecular subtypes (*BCR*::*ABL1*, *DUX4-*rearranged, *ETV6*::*RUNX1*, high hyperdiploid, low hypodiploid, *KMT2A-*rearranged, Ph-like, *PAX5-*altered, *TCF3*::*PBX1*, and *ZNF384-*rearranged) and B-other samples (Table S1) from diverse medical centers, research groups, and clinical trials networks across the United States (see STAR Methods). To identify high-confidence sites, we identified ATAC-seq peak summits using subtype-merged data and selected only loci reproducible among unmerged individual patients. Using this approach, we identified 110,468 accessible chromatin sites, on average, in each B-ALL subtype (range 71,797–142,498), with 217,240 merged sites identified in total representing the final B-ALL genomic regions of interest (Figure 1A; Table S2).

Using H3K27ac ChIP-seq data generated from a subset of 11 B-ALL patient samples, as well as primary B-ALL cell H3K27ac, H3K4me1, and H3K27me3 ChIP-seq data from the Blueprint Epigenome Consortium (https://www.blueprint-epigenome.eu/), we determined that nearly all open chromatin sites mapped to regions containing only active histone marks (H3K27ac and/or H3K4me1, 89.6%; H3K27ac, 3.3%; H3K4me1, 34%; H3K4me1+H3K27ac, 52.3%) or regions with bivalent marks, suggesting a poised chromatin state (H3K27ac and/or H3K4me1 and H3K27me3, 8.9%), compared to only 1.5% of ATAC-seq sites that mapped to regions solely harboring repressive chromatin (H3K27me3; Figure 1B). Because these histone modifications are typically found at transcriptional enhancers and promoters,^17^^,^^18^^,^^19^^,^^20^ these findings suggest that these accessible chromatin regions are B-ALL *cis*-regulatory elements implicated in gene regulation.

In most cases, these candidate *cis*-regulatory elements map within intergenic or intragenic loci with unclear gene targets. Therefore, to better inform gene connectivity, we produced chromatin looping data using promoter capture Hi-C^21^ across ten patient samples (BCR-ABL1, ETV6-RUNX1, KMT2A-rearranged, Ph-like, TCF3-PBX1, and B-other subtypes) and seven B-ALL cell lines (697, BALL1, Nalm6, REH, RS4;11, SEM, and SUP-B15) to complement B-ALL patient chromatin-accessibility profiles. Collectively, across patient samples and B-ALL cell lines we detected approximately 300,000 chromatin loops, with approximately 50% of the 217,240 chromatin-accessible regions of interest intersecting with a promoter loop, including 15,929 chromatin-accessible sites that looped to a cancer-implicated gene set (Figure 1C).^22^^,^^23^ In many instances, large domains of extensive chromatin looping are present, which with chromatin accessibility and active histone marks emphasize the gene-regulatory networks present across B-ALL patient samples (e.g., Figures 1D and 1E).

### Chromatin accessibility identifies Pro-B cell of origin for most B-ALL patient samples

**Figure 2:**
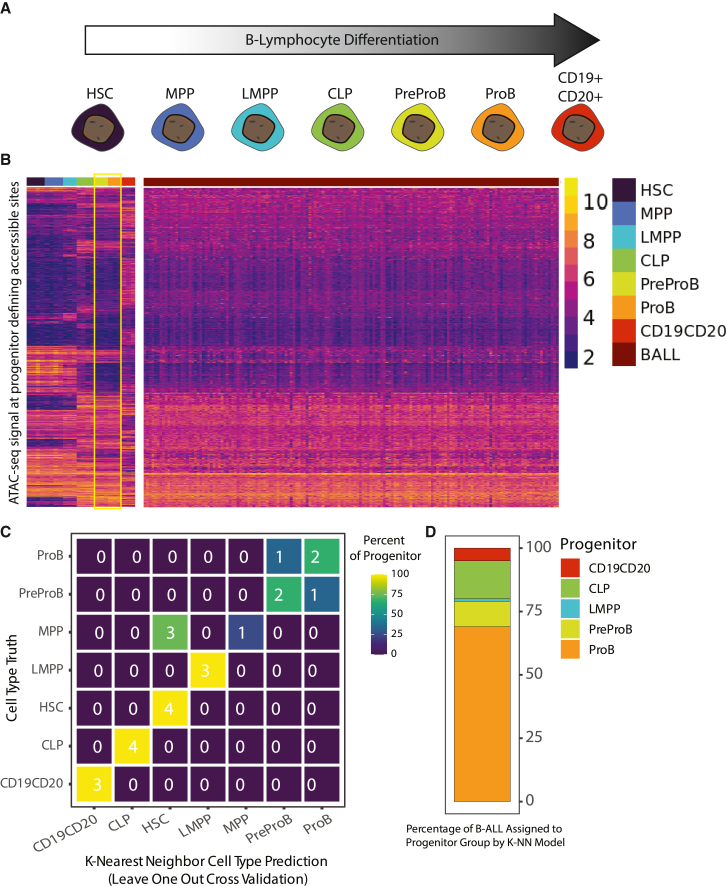
B-ALL cell type of origin defined by chromatin accessibility (A) Differentiation timeline of B cell progenitors from least differentiated to most differentiated. HSC, hematopoietic stem cell; MPP, multipotent progenitor cell; LMPP, lymphoid-primed multipotent progenitor cell; CLP, common lymphoid progenitor cell; PreProB, prePro-B cell; ProB, Pro-B cell; CD19^+^CD20^+^, B cell. (B) Heatmap using row-wise hierarchical clustering of B cell progenitor or B-ALL patient sample variance-stabilized ATAC-seq signal from DESeq2 across B cell progenitor-defining chromatin loci. B cell progenitor groups most similar to B-ALL patient samples (preProB and ProB) are outlined in yellow. (C) Confusion matrix showing number (listed) and percentage (color coded) of B cell progenitor truths and predictions for leave-one-out cross-validation of a k-nearest-neighbor classifier model. (D) Distribution of B cell progenitor classification across B-ALL patient samples using a k-nearest-neighbor classifier model trained with B cell progenitor data.

To better understand chromatin remodeling during leukemogenesis, we sought a comparison of chromatin accessibility between B-ALL and B cell progenitors. Moreover, although it is widely accepted that the B-ALL cell of origin is a B cell precursor, exactly which precursor is not always clear, particularly at the chromatin-accessibility level.^24^ To resolve this uncertainty, we examined publicly available ATAC-seq data from several human B cell progenitors^10^^,^^25^ (Figure 2A). When comparing chromatin-accessibility signal between B cell progenitor groups, we identified a set of approximately 42,344 genomic loci that demonstrate a chromatin-accessibility enrichment or depletion trend for a B cell progenitor (Figure 2B; Table S3; see also STAR Methods). We refer to these chromatin loci as B-progenitor identity loci, due to their distinct patterning across B-progenitor differentiation and likely representation of stage-specific gene-regulatory programs.

Next, we examined patient B-ALL cell chromatin accessibility across these B-progenitor identity loci. When plotting chromatin-accessibility signal as a heatmap comparing B cell progenitors and B-ALL patient samples, a high degree of similarity was observed with prePro-B cells and Pro-B cells (Figure 2B). Further, when applying the k-nearest neighbor classification model previously trained on B-progenitor identity loci, the majority of B-ALL samples were classified as either prePro-B or Pro-B (Figures 2C and 2D). However, prePro-B cells have been reported to be an extremely rare population beyond embryonic and fetal development.^25^ Overall, Pro-B cells demonstrate the most similarity to B-ALL cells at the chromatin-accessibility level when focusing specifically on B cell precursor defining loci, emphasizing this precursor B cell as a common cell of origin for B-ALL.

### Extensive differences in chromatin accessibility between B-ALL and Pro-B cells

**Figure 3:**
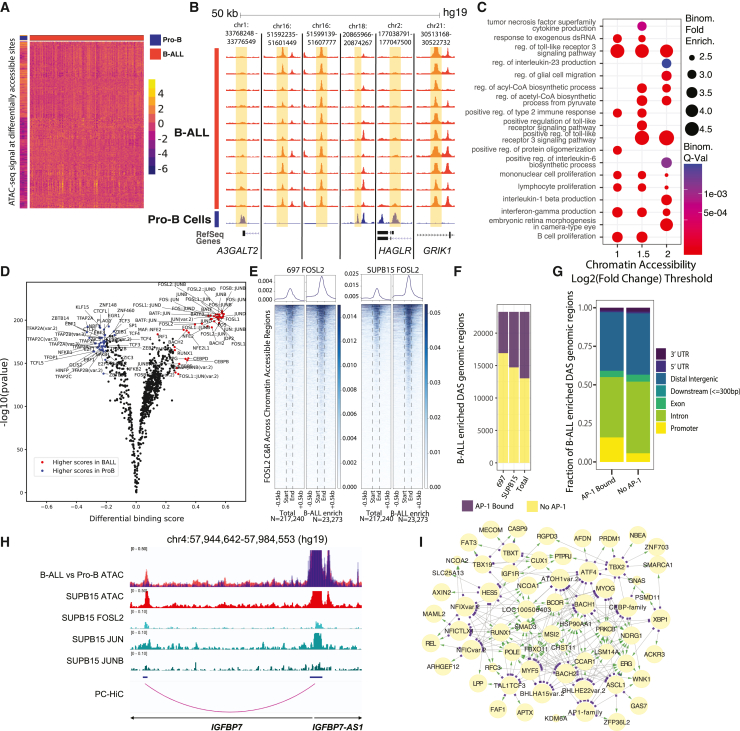
Mapping differential accessibility between B-ALL and Pro-B cells (A) Heatmap using row-wise hierarchical clustering of Pro-B cell or B-ALL patient sample variance-stabilized ATAC-seq signal as *Z* score across Pro-B cell and B-ALL-enriched DASs. DASs within heatmap are >1 or <−1 log_2_-adjusted fold change. (B) ATAC-seq signal-track examples of top Pro-B-cell-enriched DASs and B-ALL-enriched DASs on the UCSC genome browser. Flanking genomic regions are included for context. (C) Gene ontology analysis of DASs with higher accessibility in B-ALL (B-ALL enriched) at various log_2_-adjusted fold-change thresholds. All terms were significant using both binomial and hypergeometric statistical tests. (D) Differential TF footprinting between Pro-B cells and B-ALL patient samples across 217,240 B-ALL genomic regions of interest. (E) FOSL2 CUT&RUN enrichment heatmaps at all B-ALL accessible chromatin sites (on left, N = 217,240 regions) and B-ALL-enriched DASs (on right, N = 23,273 regions) in SUPB15 (left enrichment heatmap) and 697 (right enrichment heatmap) cells. Total numbers of sites are shown below each heatmap. Rows in adjacent pairs of heatmaps are unaligned. (F) Number of B-ALL-enriched DASs overlapping AP-1 TF occupancy (FOSL2, JUN, and/or JUNB) in 697 (left), SUPB15 (middle), and both B-ALL cell lines (right). Numbers of overlapping sites are shown in purple while non-overlapping sites are shown in yellow. (G) Genome annotation of B-ALL-enriched DASs with AP-1 TF occupancy (left) or that are devoid of AP-1 TF occupancy (right). (H) IGV genome browser image showing a B-ALL-enriched DAS that maps to accessible chromatin and sites of AP-1 TF occupancy in SUPB15 cells. Promoter capture Hi-C (PC-HiC) looping between the distal AP-1 occupied sites and the *IGFBP7* gene promoter is shown. B-ALL (red) and Pro-B (blue) cell ATAC-seq tracks are overlaid in the top panel. Signal tracks for FOSL2, JUN, and JUNB in SUBP15 cells are shown. (I) TF and target gene network of DASs with higher accessibility in B-ALL (B-ALL-enriched). Network is subset for top TF footprints across DASs ranked by the top mean log_2_-adjusted fold-change transcription factor footprint signal. Target genes determined with B-ALL patient origin promoter capture Hi-C are subset for a cancer-implicated gene set ranked by the top expressed genes. Network connections are colored as TFs (purple blocks) to target gene (green arrowheads) pairs. Select expansive and highly similar TF motif families are grouped (AP-1 and CEBP, AP1 family and CEBP family).

To better understand chromatin remodeling during leukemogenesis, we next compared accessible chromatin sites between B-ALL and Pro-B cells (n = 3) using DESeq2 at the 217,240 merged B-ALL chromatin-accessibility peaks and uncovered 42,661 differentially accessible chromatin sites (DASs) exhibiting lesser or greater accessibility in B-ALL samples (Figures 3A, 3B, and S1; Table S4). Ontology analysis focusing strictly on DASs with higher chromatin accessibility in B-ALL indicated an enrichment for sites associated with genes involved with Toll-like receptor signaling, interleukin production, metabolism (acetyl-coenzyme A [CoA] production), and cell proliferation (Figure 3C). Enriched ontology terms were frequently present at multiple fold-change thresholds of input B-ALL DASs (Table S5).

In addition to profiling differential chromatin accessibility, global TF binding was also compared between B-ALL and Pro-B cells. To identify differential TF binding, we performed genome-wide TF footprint profiling^12^ using 810 TF motifs comparing B-ALL patient samples and normal Pro-B cell samples across all B-ALL genomic regions of interest (217,240 regions). Differential binding scores indicated the AP-1 family of TFs (e.g., FOS, JUN) as the most prominent TFs with higher binding in B-ALL patient samples compared to normal Pro-B cells (Figure 3D). In contrast, prominent TFs with higher binding in Pro-B cells were those such as TFAP2A, KLF15, CTCFL, ZBTB14, and EBF1.

To further demonstrate AP-1 TF occupancy in B-ALL accessible chromatin sites, we performed CUT&RUN for FOSL2, JUN, and JUNB in 697 and SUB15 human B-ALL cell lines (Figures 3E and S2). Collectively, we identified 56,650 (697: FOSL2 = 66,040, JUN = 47,381, JUNB = 4,730) and 61,121 (SUPB15: FOSL2 = 106,961, JUN = 9,971, JUNB = 14,784) AP-1 binding sites in 697 and SUPB15 cells, respectively, with a final merged AP-1 region set including both 697 and SUPB15 regions of 88,650. Intersections with B-ALL accessible chromatin sites from primary cells using the merged AP-1 regions identified that 28% (61,090) of these sites were occupied by an AP-1 TF in B-ALL cell lines (41,002 sites and 19% of all B-ALL accessible chromatin sites from primary cells in 697; 45,685 sites and 21% of all B-ALL accessible chromatin sites from primary cells in SUPB15). Strikingly, our results further uncovered that 46% of DASs with higher chromatin accessibility in B-ALL (i.e., B-ALL-enriched DASs) also exhibit AP-1 TF occupancy (Figure 3F), thereby supporting the activation of AP-1 TF-associated *cis*-regulatory in B-ALL. We determined that even though most AP-1-occupied B-ALL-enriched DASs localized to promoter-distal regions of the human genome (77%), there is a 2.7-fold enrichment for AP-1 occupancy at B-ALL-enriched promoters compared to B-ALL-enriched DASs devoid of AP-1 occupancy (Figure 3G; 16% vs. 6%). Further integration of AP-1-occupied B-ALL-enriched DASs with promoter capture Hi-C in B-ALL cell lines identified target genes that were enriched for cell cycle, autophagy, and apoptotic signaling pathways (Table S6; example in Figure 3H). Select B-ALL-enriched, promoter-distal DASs predicted to be AP-1 bound by TF footprinting within B-ALL patient samples but not Pro-B cells were targeted for CRISPR-Cas9-mediated genomic deletion in B-ALL cell lines (Figure S3). Validating their role as B-ALL *cis*-regulatory elements, analysis of heterogeneous deletion pools identified effects on neighboring gene expression and cellular proliferation (Figure S4).

As an extension of our TF footprinting data, we also integrated B-ALL patient promoter capture Hi-C using the ABC enhancer algorithm to refine identification of TF-target gene relationships across top TFs and a cancer-implicated gene set.^26^ Specifically, we focused on top TF footprints within B-ALL-enriched DASs and the cancer-implicated gene targets of these DASs predicted by the ABC enhancer algorithm. Concordant with global TF footprint and AP-1 TF occupancy analyses, we identified the AP-1 family as top TFs in this network. We also identified other top TFs from TF footprinting, such as CEBP family TFs and BACH2 (Figure 3I). Other prominent top TFs include NFIC, XBP1, TBX2, and numerous basic helix-loop-helix (bHLH) class TFs (e.g., MYOG, MYF5 and HES5). Top expressed cancer-implicated gene targets for each TF converged on notable genes involved in cell signaling (*SMAD3*, *PTPRJ*), BCL6 regulation (*FBXO11*, *BCOR*), and transcriptional regulation (*RUNX1*, *ERG*, *CUX1*) (Figure 3I). Largely consistent results were further obtained using promoter capture Hi-C data from B-ALL cell lines (Figure S5). Collectively, these results highlight alterations of signaling pathways and TF-binding networks that facilitate the proliferative potential of B-ALL samples.

### Identification of subtype-enriched chromatin architecture

**Figure 4:**
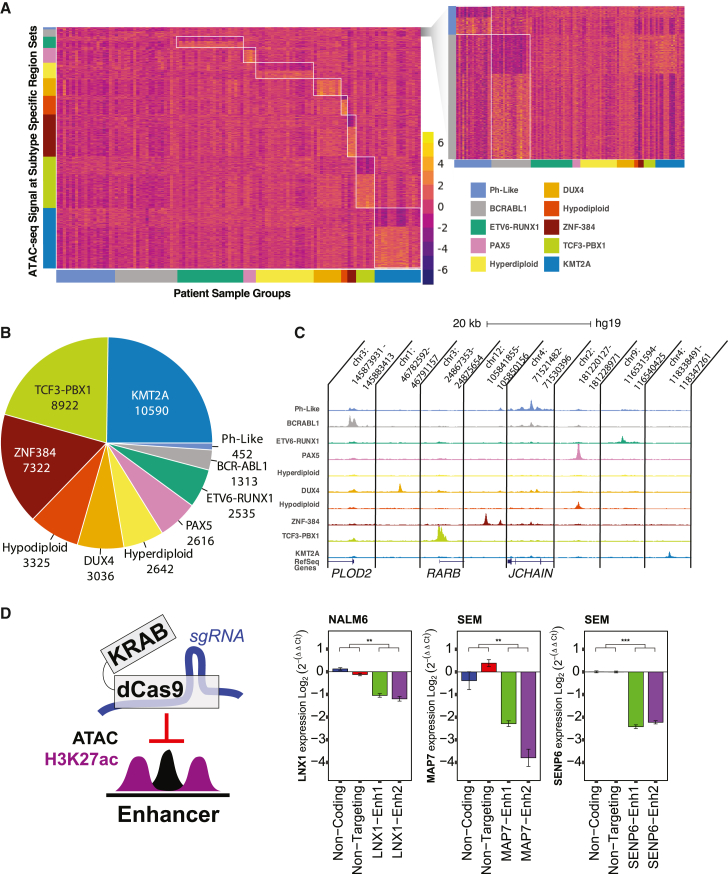
Mapping differential accessibility among B-ALL molecular subtypes (A) Heatmap of variance-stabilized ATAC-seq signal as *Z* score across subtype-enriched DASs. Enrichment patterns for each subtype DAS set are shown on vertical axis and are grouped by B-ALL subtype patient sample on the horizontal axis. Ph-like and BCR-ABL subtype-enriched DASs are expanded on the right for clarity. (B) Pie chart shows the number and percentage of subtype-enriched DASs identified. (C) ATAC-seq signal-track examples of subtype-enriched DASs on the UCSC Genome Browser. (D) dCas9-KRAB repressor targeting schematic (left) and relative transcript levels for genes associated with subtype-enriched DASs in B-ALL cell lines (right). Seventy-two hours after doxycycline (100 ng/mL) induction of SEM (*KMT2A*-rearranged) and NALM6 (*DUX4*-rearranged), B-ALL cell lines expressing doxycycline-inducible dCas9-KRAB and transduced with negative control single-guide RNAs (sgRNAs) (non-coding and non-targeting) or sgRNAs targeting subtype-enriched DASs (Enh1 and Enh2) are shown. Gene expression is normalized to the average of the two control sgRNAs (error bars denote ±standard error of the mean). Significance was calculated by a two-sample t test of combined biological replicates for both control sgRNAs versus both DAS-targeting sgRNAs. ∗∗p < 0.01; ∗∗∗p < 0.001.

To better understand chromatin accessibility within B-ALL, inter-subtype analyses were performed to identify DASs exhibiting subtype-enriched signal (henceforth referred to as subtype-enriched DASs) in ten B-ALL molecular subtypes harboring known molecular drivers (*BCR*::*ABL1*, *DUX4-*rearranged, *ETV6*::*RUNX1*, high hyperdiploid, low hypodiploid, *KMT2A-*rearranged, Ph-like, *PAX5-*altered, *TCF3*::*PBX1*, and *ZNF384-*rearranged; Figures 4A–4C). For this analysis, we compared a single B-ALL subtype cohort with all other B-ALL cell samples not belonging to that subtype in pairwise fashion covering all subtypes using DESeq2 differential analysis across the 217,240 B-ALL accessible chromatin sites from primary cells. This approach was utilized to emphasize high degrees of subtype enrichment compared to the full spectrum of chromatin-accessibility variability in the remaining sample cohort. We identified between 452 and 10,590 DASs in each B-ALL subtype, with a total of 42,753 subtype-enriched DASs identified across all ten B-ALL subtypes (log_2_ fold change >1 or <1, false discovery rate [FDR] < 0.05; Figure 4B; Table S7). We annotated subtype-enriched DASs on a subtype basis and determined that a majority of subtype-enriched DASs in each B-ALL subtype (87%, range 80%–90%) localized to promoter-distal regions of the genome (intronic and distal intergenic; Figure S6) and 43%, on average (range 39%–49%), localized to distal intergenic regions, thereby emphasizing the importance of non-genic loci in defining B-ALL chromatin heterogeneity.

To further evaluate subtype-enriched DASs, we determined whether they displayed enrichment patterns that were consistent with five established human B-ALL cell lines (697 = *TCF3*::*PBX1*, JIH5 = *ZNF384-*rearranged, Nalm6 = *DUX4-*rearranged, REH = *ETV6*::*RUNX1*, SEM = *KMT2A-*rearranged, and SUPB15 = *BCR*::*ABL1*). Concordant with DASs in patient samples, subtype-enriched DASs exhibited the strongest (BCR-ABL, *DUX4-*rearranged, *ETV6*::*RUNX1*, *KMT2A-*rearranged) or second strongest (*TCF3*::*PBX1*) accessibility in the concordant cell line that was representative of that subtype (Figure S7). These data suggest that B-ALL cell lines exhibit chromatin accessibility that is largely consistent with the primary B-ALL cell sample from the corresponding subtype.

To further determine functional effects on gene expression, we integrated subtype-enriched DASs with differentially expressed genes (DEGs) uniquely upregulated (log_2_ fold change > 1, FDR < 0.05) in each of the ten B-ALL molecular subtypes to determine whether they were enriched near DEGs. We identified a statistically significant enrichment of subtype-enriched DASs near upregulated DEGs in nine of ten subtypes compared to total expressed genes in the corresponding subtype and uncovered a strong statistical trend in Ph-like B-ALL (Kolmogorov-Smirnov test, p = 0.053; Figure S8). We additionally selected several top differential DAS genomic regions for targeting with the CRISPR interference (CRISPRi) dCas9-KRAB repressor as a test of their effects on neighboring gene expression (Figures 4D and S9). Putative *cis*-regulatory elements were targeted within a B-ALL cell line context corresponding to the origin of the B-ALL subtype DASs (Nalm6 = *DUX4-*rearranged, SEM = *KMT2A-*rearranged). These select DAS regions each demonstrated repression of the corresponding nearby genes (*LNX1*, *MAP7*, *SENP6*) when targeted with a dCas9-KRAB repressor, indicating a genuine *cis*-regulatory element (Figure 4D). Consequently, these data support the role of subtype-enriched DASs in gene regulation and gene activation and further suggest that differences in chromatin accessibility contribute to transcriptomic differences among B-ALL subtypes.^3^^,^^4^ Collectively, these results highlight extensive open chromatin heterogeneity among B-ALL molecular subtypes.

### Mapping transcription factor drivers and gene-regulatory networks in B-ALL subtypes

**Figure 5:**
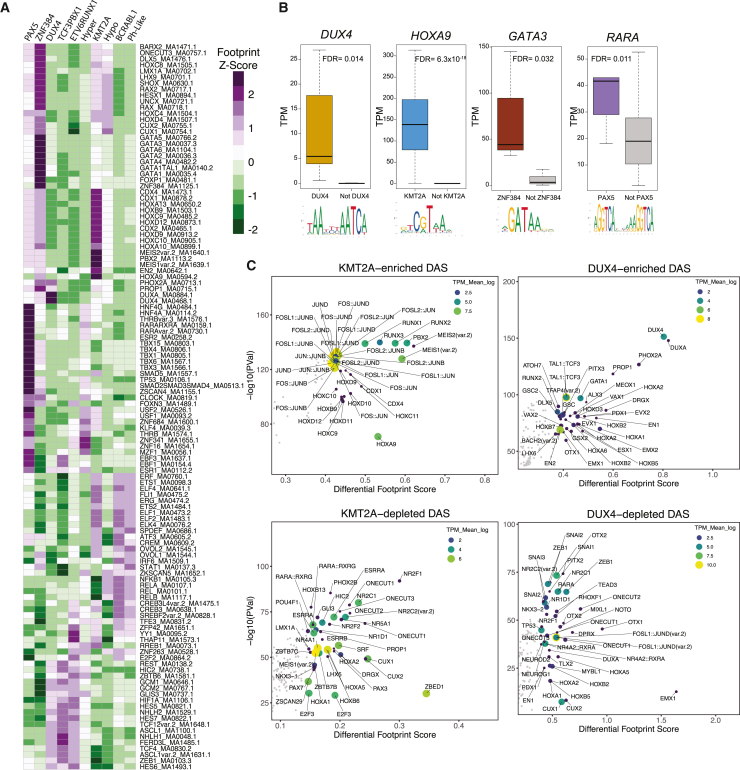
TF footprinting and gene-regulatory networks identify key TF drivers in B-ALL subtypes (A) Heatmap list of the topmost consistently differential TF footprints between all pairwise subtype-subtype comparisons (y axis; labeled to the right of the heatmap as TF motif identifiers) enriched in ten B-ALL subtypes (x axis; labeled on top of heatmap as *Z* score of differential TF footprint signal output by TOBIAS). (B) RNA-seq transcripts per million (TPM) expression of key TFs with subtype-enriched footprints that are also upregulated in the corresponding subtype (colored) versus all other subtypes (gray). DESeq2 differentially expressed gene FDR significance values are provided. (C) Top TF footprints at DASs that are enriched (top) or depleted (bottom) in *KMT2A*-rearranged (left) and *DUX4*-rearranged (right) B-ALL. Differential footprint score between merged subtype patient samples and non-subtype patient samples is provided on the x axis, and TF footprint significance is provided on the y axis. Higher differential footprint scores indicate higher binding in the merged subtype group compared to all other merged non-subtype samples. TPM transcript abundance of associated TF transcript in the merged subtype group is shown as both color and size of points.

We performed TF footprint profiling for 810 TF motifs across all B-ALL chromatin-accessibility sites (N = 217,240) using merged ATAC-seq signal from ten B-ALL subtypes with known molecular drivers to identify subtype-enriched TF drivers. TF footprint profiling^12^ identified between 4,303,155 and 5,441,937 bound motifs in each B-ALL subtype, with 49,402,067 TF footprints at 815,992 unique genomic loci identified across all subtypes. Using these data, we next identified key TF footprints that were enriched in each subtype (i.e., subtype-enriched TF footprints) by calculating differential footprint scores between every subtype-subtype pair for each TF motif. The top median differential motif scores for each subtype were selected as subtype-enriched TF footprints. This approach was utilized to emphasize differential TF footprint motifs that were consistent and distinct for each subtype rather than repetitive global trends (Figure 5A). Notably, subtype-enriched TF footprints were identified for recognized TF drivers such as DUX4 in *DUX4-*rearranged ALL and ZNF384 in *ZNF384-*rearranged ALL. We also identified HOX family TFs (HOXA9, HOXB9, HOXC9, and HOXD9) in *KMT2A-*rearranged ALL, GATA family TFs (GATA2, GATA3, GATA4, GATA5, and GATA6) in *ZNF384-*rearranged ALL, and nuclear receptor family TFs (ESR1, ESR2, RARA, and THRB) in *PAX5-*altered ALL that all had strong subtype-enriched TF footprints.

Because DNA consensus motifs can be highly redundant within TF families, we integrated subtype-enriched TF footprints with DEGs uniquely upregulated in each subtype to identify candidate TFs from these TF families that are upregulated in the corresponding B-ALL subtype. This analysis identified *HOXA9* and *HOXC9*, *RARA*, and *GATA3* as upregulated genes in *KMT2A-*rearranged, *PAX5-*altered, and *ZNF384-*rearranged subtypes, respectively (Figure 5B). In addition, *DUX4* (*DUX4-*rearranged) and *MEIS1* (*KMT2A-*rearranged) were also identified as upregulated TF genes with subtype-enriched TF footprints (Figure S10).

To determine whether these upregulated TFs promote unique chromatin-accessibility landscapes among B-ALL subtypes, we also performed TF footprinting on subtype-enriched DASs by comparing differential footprint scores between each B-ALL subtype group and non-subtype patient sample group (Figures 5C and S11). These data also supported a role of DUX4 in *DUX4-*rearranged ALL, ZNF384 and GATA3 in *ZNF384-*rearranged ALL, and HOXA9 and MEIS1 in *KMT2A-*rearranged ALL in the generation of subtype-specific chromatin landscapes (Figures 5C and S11). Additionally, our subtype versus non-subtype findings for *ETV6*::*RUNX1* further confirm the importance of factors binding GGAA DNA-sequence repeats (EWSR1-FLI1, MA0149.1) as previously characterized for the *ETV6*::*RUNX1* subtype^27^ (Figure S11). In complement to analysis of subtype-enriched DASs, we also examined TF footprints among subtype-depleted DASs by again comparing each B-ALL subtype group and non-subtype group. Transcriptional repressors such as ZNF135, ZNF263, ZEB1, and ZEB2 had higher footprint scores across subtype-depleted DASs for multiple subtypes, suggesting a common set of TFs promoting subtype-depleted DASs (Figure 5C and Data S1).

### Predictive potential of B-ALL subtype-enriched DASs

**Figure 6:**
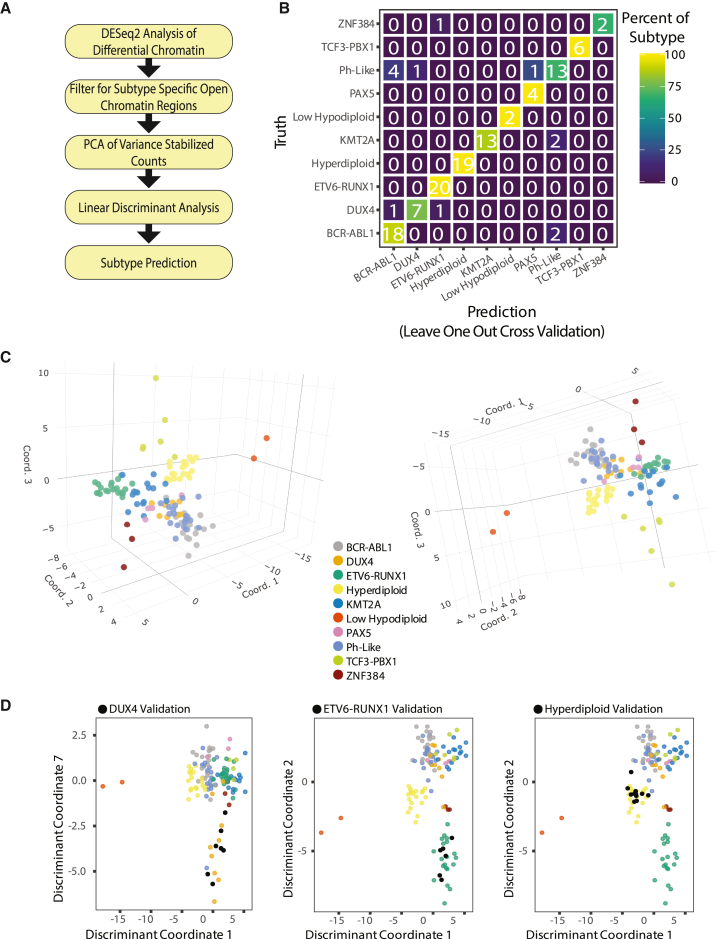
Classification model accurately predicts B-ALL subtypes (A) Flow chart outlines process for PCA-LDA classification of B-ALL subtypes. (B) Confusion matrix showing number (listed) and percentage (color coded) of B-ALL subtype truths and predictions for leave-one-out cross-validation. (C) Three-dimensional plots showing clustering of B-ALL subtypes utilizing select dimensions from the LDA model. (D) LDA clustering of validation cohort B-ALL patient samples with training samples after processing with the classification algorithm. Validation samples for each subtype group are shown in black. Data are provided for DUX4-rearranged (left), *ETV6*::*RUNX1* (center), and hyperdiploid (right) B-ALL.

We determined how well chromatin accessibility can predict B-ALL subtypes by constructing a stepwise principal component analysis-linear discriminant analysis (PCA-LDA) classification model using the 42,753 subtype-enriched DAS ATAC-seq read count matrix as initial input across ten B-ALL subtypes harboring known molecular drivers (outlined in Figure 6A). Notably, the constructed classification model was tested with leave-one-out cross-validation at an accuracy of 89%. The most common failure was incorrect classification of *BCR*::*ABL1* and Ph-like subtypes (Figure 6B), as has been observed with other ALL classification algorithms.^28^ Taking this into account by grouping *BCR*::*ABL1* and Ph-like subtype samples into a common class yielded a recalculated cross-validation accuracy of 91%. Visualization of B-ALL subtype separations using select dimensions output by the LDA model demonstrates distinct groupings of related subtypes emphasizing classification-model performance (Figure 6C).

As a further validation of our classification model, we applied the classification algorithm to a separate ATAC-seq validation cohort comprising 24 B-ALL patient samples of known subtype covering *ETV6*::*RUNX1*, *DUX4*-rearranged and hyperdiploid subtypes from our previous work.^29^ The classification model was able to assign the correct subtype with 100% accuracy among the 24 B-ALL patient samples in the validation set. Classification-model performance was visualized by projecting the validation cohort onto the original training model LDA dimensions, yielding a distinct clustering of training samples with validation samples (Figure 6D). Collectively, these data support the utility of chromatin structure and subtype-enriched DASs in B-ALL subtype classification.

### Mapping inherited DNA-sequence variants that impact chromatin accessibility

**Figure 7:**
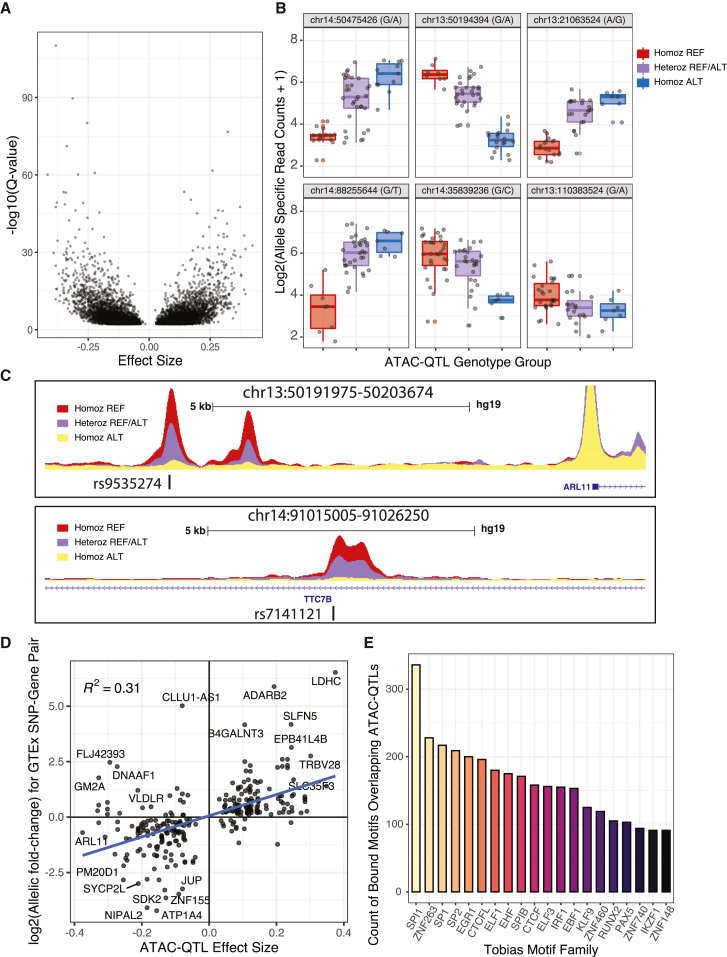
Identification of ATAC-QTLs impacting chromatin accessibility (A) ATAC-QTL effect size (x axis) and significance (y axis) is plotted for all significant ATAC-QTLs (FDR < 0.1). (B) Examples of allele-specific effects on ATAC-seq read count at ATAC-QTLs between samples from the three genotype groups. Homoz_REF, homozygous reference allele; Heteroz_REFALT, heterozygous; Homoz_ALT, homozygous alternative allele. (C) UCSC browser ATAC-seq signal tracks of merged BAM files from patients with distinct genotypes at *ARL11* (top panel) and *TTC7B* (bottom panel) gene loci. ATAC-QTLs are marked by an asterisk. Homoz_REF, homozygous reference allele; Heteroz_REFALT, heterozygous; Homoz_ALT, homozygous alternative allele. (D) Scatterplot of effect size for SNPs significant as both ATAC-QTLs (x axis) and GTEx lead eQTL (y axis). (E) Abundance of top TF-bound motifs overlapping ATAC-QTLs. TF-bound motifs on the x axis were grouped into families (TOBIAS Motif Family), which represents highly similar TF motifs based on sequence grouped into motif families via TOBIAS motif clustering.

To determine how germline variation impacts chromatin accessibility, we identified chromatin-accessibility ATAC-QTLs in a subset of 69 patient samples with available SNP genotyping information and allele-specific ATAC-seq read counting using RASQUAL.^30^ In total, 9,080 ATAC-QTLs were identified representing both directionalities, with reference or alternative alleles increasing chromatin accessibility (FDR < 0.1; Figure 7A; Table S8). Manual quantification and scaling of allele-specific read counts for select ATAC-QTLs identified with RASQUAL demonstrated a clear concordance and directionality among individual patient samples classified into genotype groups (Figure 7B). Visual inspection of merged read counts from patient samples grouped into reference allele homozygote, heterozygote, or alternative allele homozygote for select ATAC-QTLs further supports the high-quality nature of identified ATAC-QTLs (Figure 7C). We further determined that 218 ATAC-QTLs were also lead expression QTL (eQTL) SNPs when compared to gene-tissue expression (GTEx) eQTLs^31^ from relevant tissues (blood and lymphoblastoid cells), with 85% also concordant for allele over-representation directionality (Figure 7D; Table S9). ATAC-QTLs were also compared with inherited genome-wide association study variants for ALL disease susceptibility, which identified rs3824662 (*GATA3*)^32^ and rs17481869 (2p22.3)^33^ as ATAC-QTLs that were associated with the risk of developing B-ALL. Further supporting the validity of our methodology, rs3824662 was also identified as an ATAC-QTL in ALL PDX samples,^34^ and we functionally validated differential allele-specific activity for rs17481869 in multiple B-ALL cell lines (Figure S11).

To infer the impact of TF binding in control of chromatin accessibility at ATAC-QTLs, we overlapped ATAC-QTL loci with TF motifs determined as TF-bound by footprint profiling.^12^ Nearly one-third (28.8%; 2,615/9,080 ATAC-QTLs) of these ATAC-QTLs overlapped a TF-bound motif footprint across multiple B-ALL subtypes, suggesting that most ATAC-QTLs do not have a clear TF-binding mechanism regarding how they impact chromatin accessibility. Analysis of bound TF motif footprint prevalence at ATAC-QTLs identified several ETS family TFs (EHF, ELF3, SPI1/PU.1, and SPIB), zinc-finger TFs (ZNF263, ZNF460, ZNF740, and ZNF148), and CTCF as the most altered motifs leading to differences in chromatin accessibility between alleles (Figure 7E). Notably, we also identified PAX5 and IKZF1, which have known roles in B cell development and leukemogenesis.^35^^,^^36^^,^^37^^,^^38^ Collectively, these data identify inherited DNA-sequence variants contributing to chromatin heterogeneity among B-ALL subtypes and indicate specific TFs of interest for further exploration of ATAC-QTLs.

## Discussion

Our study provides a large-scale examination of chromatin accessibility in the B-ALL genome across an expansive set of B-ALL subtypes. We further integrated these data with ChIP-seq histone modification enrichment in primary B-ALL cells and three-dimensional chromatin looping data using promoter capture Hi-C in multiple patient samples and B-ALL cell lines. Our data demonstrate that most regions of chromatin accessibility harbor activating chromatin marks consistent with *cis*-regulatory elements involved in gene regulation, and we further confirmed direct looping to gene promoters for approximately 50% of accessible chromatin sites. However, this does not rule out more transient chromatin looping interactions difficult to detect by current chromatin conformation capture genomic techniques.

Extensive epigenomic reprogramming was uncovered between B cell progenitors and B-ALL, and cell-of-origin analyses identified Pro-B cells as the most common cell of origin. Our comparison of B-ALL and Pro-B cell chromatin accessibility suggests epigenomic reprogramming that is, in part, associated with AP-1 TF occupancy. We further identify disruptions to normal B cell function through the activation of Toll-like receptor signaling and interleukin production. Acetyl-CoA synthesis was also identified as an enriched gene ontology term when comparing B-ALL and Pro-B cells. Metabolic alterations in cancer are well known, particularly acetyl-CoA synthesis alterations, which have been previously reported in cancer.^39^

We further examined accessible chromatin landscapes among diverse molecular subtypes of B-ALL. Collectively, we identified 42,753 subtype-enriched DASs, which strikingly represent 20% of analyzed accessible chromatin sites across a pan-subtype B-ALL genome. Subtype-enriched DASs were enriched near upregulated DEGs in the corresponding subtype, supporting their role in gene activation. Moreover, comparisons between subtype-enriched DASs and chromatin-accessibility data from cell lines identified largely consistent patterns. We further identified candidate TFs that exhibited strong subtype specificity through TF footprinting analyses and validated some of these findings using transcriptomic data from primary B-ALL cells. Collectively, these analyses highlighted a putative role for HOXA9 and MEIS1 in *KMT2A-*rearranged ALL, GATA3 in *ZNF384-*rearranged ALL, and RARA in *PAX5-*altered B-ALL. We further confirmed the previously reported roles of DUX4 and ZNF384 in *DUX4-*rearranged and *ZNF384-*rearranged ALLs, respectively, and our more focused analysis of subtype-enriched DASs confirmed many of these TF hits. Concordant with our findings, previous studies have identified the co-upregulation of HOXA9 and MEIS1 in *KMT2A-*rearranged leukemias and further support that these TFs are key drivers of leukemogenesis.^40^^,^^41^^,^^42^ Our identification of numerous HOX TFs with enriched footprints in *KMT2A-*rearranged ALL is also consistent with observations of HOX gene dysregulation in this subtype.^43^ Further supporting our results, ZNF384 fusion proteins in *ZNF384-*rearranged ALL are known to upregulate *GATA3* expression.^44^^,^^45^ Although a direct role for RARA in *PAX5-*altered B-ALL has not been established, previous work has identified *PAX5* as a target gene of the PLZF-RARA fusion protein in acute promyelocytic leukemia.^46^ Moreover, both *RARA* and *PAX5* genes can form fusions with *PML* in acute promyelocytic leukemia^47^ and ALL,^48^ respectively. While *PAX5-*altered ALL has not been well connected to RARA nuclear receptor signaling, there has been previous work treating IKZF1-mutated BCR-ABL1 ALL with RARA and RXR agonists that suppressed a self-renewal phenotype.^49^ Collectively, these data warrant further investigation of RARA and RXR signaling in *PAX5-*altered ALL.

In addition to key subtype-enriched TF footprints identified within accessible chromatin sites, we also assessed subtype-depleted DASs and identified numerous TFs that have been shown to act as repressors, such as ZNF135, ZNF263, ZEB1, and ZEB2. Intriguingly, several studies have supported a role for these repressors in cancers such as neuroblastoma and AML demonstrating enhanced tumorigenic phenotypes.^50^^,^^51^ Our observations support a more detailed examination of these repressive TFs in the occlusion of accessible chromatin sites in B-ALL.

Supporting the utility of chromatin accessibility in B-ALL classification, subtype-enriched DASs predicted subtypes with 89% accuracy. As a comparison to chromatin accessibility, transcriptional profiling using ALLSorts correctly assigned B-ALL subtypes with 92% accuracy.^28^ However, this RNA-sequencing (RNA-seq) dataset included over 1,223 transcriptomes from 18 subtypes, representing a considerably larger dataset for model development. We therefore suspect that additional chromatin-accessibility profiling across more B-ALL subtypes and increased sample sizes will lead to even better subtype prediction that will rival transcriptomic profiling and, importantly, incorporate intergenic heterogeneity that can elucidate *cis*-regulatory drivers of B-ALL leukemogenesis.

To identify the role of inherited DNA-sequence variation on the B-ALL chromatin landscape, we mapped over 9,000 ATAC-QTLs (FDR < 0.1). A large subset of ATAC-QTLs mapped to TF footprints and was concordant in allelic biases with GTEx lead eQTLs. We additionally identified several key TFs that appear to impact ATAC-QTL signal and chromatin accessibility, including a subset known to impact B cell development and leukemogenesis, such as PAX5 and IKZF1.^35^^,^^36^^,^^37^^,^^38^ In addition, we identified ZNF263 as a top hit, which is consistent with our demonstration of enrichment for this transcriptional repressor at subtype-depleted DASs. Further validating our analysis, we functionally validated a variant (rs17481869; 2p22.3) associated with susceptibility to ALL.^33^ Collectively, this analysis suggests that chromatin accessibility is additionally modified by inherited DNA-sequence variation, thereby further contributing to increased chromatin heterogeneity in B-ALL.

Overall, our data support pronounced changes in chromatin accessibility between B-ALL and precursor B cells as well as among B-ALL subtypes. Our results further support the role of diverse TFs and inherited genetic variants in modulating and promoting differences in chromatin accessibility among B-ALL subtypes. Ultimately, these diverse chromatin architectures contribute to unique gene-regulatory networks and transcriptional programs. Our work therefore provides a valuable resource to the cancer genomics research community and can be further used to better understand biological as well as clinical differences among B-ALL subtypes.

### Limitations of the study

Our study provides a valuable resource for the genomics and cancer research communities. Because this work represents a resource, a key limitation is the lack of mechanistic insights with extensive experimental validation. Indeed, many of our analyses, such as comparisons with B-ALL cell lines and B-ALL RNA-seq datasets, were performed to validate the accuracy and robustness of our ATAC-seq data. Our identification of Pro-B cells as the most common cell of origin was identified in mouse models,^52^ and a role for AP-1 TFs in B-ALL was also described in *KMT2A*-rearranged ALL.^53^ We also identified known B-ALL molecular drivers (e.g., DUX4 and ZNF384 in *DUX4*-rearranged and *ZNF384*-rearranged ALL, respectively) and previously documented gene-regulatory alterations in B-ALL (e.g., *HOXA9* and *MEIS1* activity in KMT2A-rearranged ALL). Collectively, these data support the validity of our ATAC-seq dataset. We further mapped key TF-target gene interactions that are enriched in B-ALL compared to progenitor B cells. Additional work, including the generation of H3K27ac ChIP-seq and promoter capture Hi-C data in Pro-B cells and other B cell progenitors, is required to establish the extent of regulatory rewiring in B-ALL by these TFs. We also identified multiple additional TFs, including transcriptional repressors, in driving unique chromatin architectures among B-ALL subtypes. Additional experimentation is likewise required to validate the roles for these TFs in maintaining or establishing chromatin architecture and in subtype biology. Our classification model was able to predict molecular subtypes with high accuracy. We believe that additional ATAC-seq datasets, including data from rarer subtypes not interrogated in this study, will improve the accuracy of chromatin accessibility in discriminating B-ALL molecular subtypes. Further functional experimentation is also required to validate the effects of ATAC-QTLs on TF-binding events and neighboring gene expression and to determine whether these inherited genetic variants are associated with additional B-ALL cellular phenotypes or even clinical phenotypes in patients.

## STAR★Methods

### Key resources table

**Table:** 

REAGENT or RESOURCE	SOURCE	IDENTIFIER
**Antibodies**		

H3K27Ac Rabbit pAb	Active Motif	#39133; RRID:AB_2561016
Fra2 (D2F1E) Rabbit mAb	Cell Signaling	#19967, Lot: 2; RRID:AB_2722526
JUN/c-Jun CUTANA™ CUT&RUN Antibody	Epicypher	13-2019, Lot: 22070001-85
JunB (C37F9) Rabbit mAb	Cell Signaling	#3753, Lot: 2; RRID:AB_2130002
CUTANA™ Rabbit IgG CUT&RUN Negative Control Antibody	Epicypher	13–0042; RRID:AB_2923178

**Biological samples**		

Patient B-ALL cell samples	St. Jude Children’s Research Hospital	https://Stjude.org
Patient B-ALL cell samples	ECOG-ACRIN Cancer Research Group	https://ecog-acrin.org/
Patient B-ALL cell samples	The Alliance for Clinical Trials in Oncology	https://allianceforclinicaltrialsinoncology.org/main/
Patient B-ALL cell samples	MD Anderson Cancer Center	https://www.mdanderson.org/
Patient B-ALL cell samples	Cook Children’s Medical Center	https://www.cookchildrens.org/
Patient B-ALL cell samples	Lucile Packard Children’s Hospital	https://www.stanfordchildrens.org/en/lucile-packard-childrens-hospital
Patient B-ALL cell samples	The University of Chicago	https://www.uchicago.edu
Patient B-ALL samples	Novant Health Hemby Children’s Hospital	https://www.novanthealth.org/locations/medical-centers/hemby-childrens-hospital/
Patient B-ALL cell samples	Children’s Hospital of Michigan	https://www.childrensdmc.org/

**Critical commercial assays**		

Cutana CUT&RUN kit v3.0	Epicypher	14–1048
Human promoter capture Hi-C kit	Arima	A510008, A303010, A302010
Illumina Tagment DNA Enzyme and Buffer Large Kit	Illumina	20034198
Dual Luciferase Reporter Assay System	Promega	E1960
PowerPlex Fusion STR	Promega	DC2402
MycoScope PCR detection kit	Genlantis	MY01050

**Deposited data**		

Progenitor B cell ATAC-seq	Corces et al.^10^; O’Byrne et al.^25^	GEO: GSE122989, GSE74912
B-ALL cell line ATAC-seq and H3K27ac ChIP-seq tracks associated with dCas9-KRAB targeting	Kodgule et al.^27^	GEO: GSE186942
B-ALL cell sample histone modification ChIP-seq datasets (H3K27ac, H3K4me1 and H3K27me3)	Blueprint Epigenome Consortium	https://www.blueprint-epigenome.eu/
Patient B-ALL samples (validation cohort)	Diedrich et al.^29^	GEO: GSE161501
Patient B-ALL cell sample ATAC-seq	This paper	GEO: GSE211631
Patient B-ALL cell sample ChIP-seq	This paper	GEO: GSE211631
Patient B-ALL cell sample promoter capture Hi-C	This paper	GEO: GSE211631
B-ALL cell line promoter capture Hi-C	This paper	GEO: GSE211631
B-ALL cell line AP-1 factor CUT&RUN	This paper	GEO: GSE211631
Patient B-ALL cell sample RNA-seq data	St. Jude Children’s Research Hospital	St. Jude Cloud
Patient B-ALL cell sample Variant Call Frequency (VCF) genotyping data	St. Jude Children’s Research Hospital	St. Jude Cloud

**Experimental models**: **Cell lines**		

697 B-ALL cells	DSMZ	ACC 42; RRID:CVCL_0079
BALL1 B-ALL cells	DSMZ	ACC 742; RRID:CVCL_1075
Nalm6 B-ALL cells	ATCC	CRL-3273; RRID:CVCL_0092
REH B-ALL cells	ATCC	CRL-8286; RRID:CVCL_1650
RS4; 11 B-ALL cells	ATCC	CRL-1873; RRID:CVCL_0093
SEM B-ALL cells	DSMZ	ACC 546; RRID:CVCL_0095
SUP-B15 B-ALL cells	ATCC	CRL-1929; RRID:CVCL_0103
JIH-5 B-ALL cells	DSMZ	ACC 788; RRID:CVCL_EQ76

**Oligonucleotides**		

CRISPR-Cas9 deletion DNA oligo sequences	This paper	See Table S10
dCas9-KRAB CRISPRi DNA oligo sequences	This paper	See Table S11
rs17481869 luciferase assay DNA oligo sequences	This paper	See Table S12

**Recombinant DNA**		

TRE3-KRABdCas9-IRES-GFP	Fulco et al.^54^	Addgene #85556
sgOpti	Fulco et al.^54^	Addgene #85681
pLVX-EF1alpha-Tet3G	Clontech	631359
pGL4.23	Promega	E841A
pRL-TK renilla	Promega	E2231

**Software and algorithms**		

Arima CHiC pipeline (v1.4)	Arima Genomics	https://github.com/ArimaGenomics/CHiC
FlashFry	McKenna et al.^55^	https://github.com/mckennalab/FlashFry
ChIPseeker (v1.30.3)	Yu et al.^56^	https://bioconductor.org/packages/release/bioc/html/ChIPseeker.html
DESeq2 (v1.34.0)	Love et al.^57^	https://bioconductor.org/packages/release/bioc/html/DESeq2.html
Pheatmap (v1.0.12)	Kolde^58^	https://github.com/raivokolde/pheatmap
Clusterprofiler (v4.2.2)	Yu et al.^59^	https://bioconductor.org/packages/release/bioc/html/clusterProfiler.html
ATACseqQC (v1.18.1)	Ou et al.^60^	https://bioconductor.org/packages/release/bioc/html/ATACseqQC.html
ggplot2 (v3.3.6)	Wickham^61^	https://github.com/tidyverse/ggplot2
Michigan Imputation Server (v1.6.5)	Das et al.^62^	https://imputationserver.sph.umich.edu/
RASQUAL (v1.1)	Kumasaka et al.^30^	https://github.com/natsuhiko/rasqual
TrimGalore (v0.6.6)	Krueger^63^	https://github.com/FelixKrueger/TrimGalore
Bowtie2 (v2.2.9)	Langmead and Salzberg^64^	http://bowtie-bio.sourceforge.net/bowtie2/index.shtml
Samtools (v1.2)	Li et al.^65^	http://samtools.sourceforge.net/
Picard (v1.141)	Broad Institute^66^	https://github.com/broadinstitute/picard
MACS2 (v2.1.1)	Zhang et al.^67^	https://github.com/macs3-project/MACS/wiki/Install-macs2
Bedtools (v2.30.0)	Quinlan and Hall^68^	https://github.com/arq5x/bedtools2
nondetects	Sherina et al.^69^	https://www.bioconductor.org/packages/release/bioc/html/nondetects.html
Primer-Blast	Ye et al.^70^	http://www.ncbi.nlm.nih.gov/tools/primer-blast
CHiCAGO (v1.22.0)	Freire-Pritchett et al.^71^	https://www.bioconductor.org/packages/devel/bioc/vignettes/Chicago/inst/doc/Chicago.html
HiCup (v0.8.0)	Wingett et al.^72^	https://www.bioinformatics.babraham.ac.uk/projects/hicup/
nextflow-core cutandrun pipeline (v2.0.0)	Meers et al.^73^	https://github.com/nf-core/cutandrun
R (v4.1.0)	R Core Team^74^	https://www.R-project.org/
TOBIAS (v0.12.11)	Bentsen et al.^12^	https://github.com/loosolab/TOBIAS
Cutadapt (v1.18)	Martin^75^	https://github.com/marcelm/cutadapt/
Fastqc (v0.11.9)	Andrews^76^	https://www.bioinformatics.babraham.ac.uk/projects/fastqc/
Deeptools (v3.5.0)	Ramirez et al.^77^	https://deeptools.readthedocs.io/en/develop/index.html
ABC-Enhancer-Gene-Prediction	Fulco et al.^26^	https://github.com/broadinstitute/ABC-Enhancer-Gene-Prediction
GREAT	McLean et al.^78^	https://great.stanford.edu/great/public/html/
Custom code	This paper	https://github.com/Savic-Lab/B-ALL_Chromatin_Landscape/and https://doi.org/10.5281/zenodo.10018584

### Resource availability

#### Lead contact

Further information and requests for resources, including associating patient ATAC-seq data with other functional genomic data (e.g., RNA-seq) from the same patient biospecimens that are available on St. Jude Cloud (https://www.stjude.cloud/), should be directed to and will be fulfilled by the lead contact, Daniel Savic (daniel.savic@stjude.org)

#### Materials availability

Cell lines and plasmids generated in this study are available upon request from the lead contact.

#### Data and code availability

ATAC-seq, H3K27ac ChIP-seq, CUT&RUN-seq, and promoter capture Hi-C from patient or cell line origin biospecimens have been deposited to NCBI Gene Expression Omnibus (GEO: GSE211631). Key code used in the analysis of data is available at: https://github.com/Savic-Lab/B-ALL_Chromatin_Landscape and https://doi.org/10.5281/zenodo.10018584. In the event of lead contact unavailability, guidance for acquiring additional data associated with patient samples can be requested from Kristine Crews (kristine.crews@stjude.org).

### Experimental model and study participant details

#### Human subjects

Patient samples were obtained from: St. Jude Children’s Research Hospital (Memphis, Tennessee), ECOG-ACRIN Cancer Research Group, The Alliance for Clinical Trials in Oncology, MD Anderson Cancer Center (Houston, Texas), Cook Children’s Medical Center (Fort Worth, Texas), Lucile Packard Children’s Hospital (Palo Alto, California), The University of Chicago (Chicago, Illinois), Novant Health Hemby Children’s Hospital (Charlotte, North Carolina) and Children’s Hospital of Michigan (Detroit, Michigan). A list of patient biospecimens is provided in Table S1. All patients or their legal guardians provided written informed consent. The use of these samples was approved by the institutional review board at St. Jude Children’s Research Hospital. Sample size estimation was not performed prior to analyses. Patient samples were allocated to experimental groups (general B-ALL or B-ALL subtypes) based upon a combination of immunophenotype, cytogenetic and RNA transcript profiling. Detailed demographics of human patient samples were not available to incorporate into analyses. Differential chromatin accessibility loci localized to sex chromosomes should be utilized with caution.

#### Cell lines

ALL cell lines utilized in this study (SUPB15, 697, BALL1, SEM, REH, Nalm6, RS411, JIH5) were cultured in RPMI 1640 medium (GibCo 2492873) supplemented with 1% GlutaMAX (GibCo 35050061) 10% FBS and maintained at a target cell density in the range of 1 – 3x10^6^ cells/mL. JIH5 cells were cultured in the same medium containing 20% FBS. Cell lines were authenticated with PowerPlex Fusion STR (Promega) profiling and screened for mycoplasma using the MycoScope PCR detection kit (Genlantis).

### Method details

#### ATAC-seq

ATAC-seq using the Fast-ATAC^10^ protocol was performed on 10,000 fresh primary ALL cells. Briefly, 10,000 cells were pelleted in a 1.5 mL Eppendorf low-bind sample tube (#022431021) and resuspended in 25 μL of transposase mix (25 μL TD buffer, 2.5 μL TDE1, 0.5 μL 1% digitonin, and 22 μL nuclease free water; see key resources table). Transposase reactions were incubated in 1.5mL Eppendorf low-bind sample tubes at 37°C on a thermomixer set at 300 rpm. DNA was then purified using the MinElute PCR Purification Kit and eluted in 10 μL of elution buffer. Libraries were indexed and amplified using NEBNext 2x PCR master mix (New England Biolabs, M0541L). Amplified DNA libraries were sequenced on a Nova-seq 6000 or NovaSeq X+ (Illumina) using 150bp paired-end sequencing. Adapter trimming was performed using TrimGalore (v0.6.6)^63^ with command options “--fastqc --paired”. Read mapping was performed using Bowtie2 (v2.2.9)^64^ and hg19 genome index with custom command options “-X 2000 -S”. Read quality filtering was performed with Samtools^65^ with the “view” command and options “-q 20 -b”. Sorting was performed with Picard (v1.141)^66^ using the “SortSam” command and options “SORT_ORDER = coordinate”. Mitochondrial reads were removed using Samtools “view” command combined with command line filtering, “samtools view -h bam | awk '{if($3 ! = "chrM"){print $0}}' | samtools view -b - > bam”. Peak calling was performed with MACS2^67^ (v2.1.1 using command and options “macs2 callpeak -t bam -f BAMPE -g hs --nomodel --extsize 200 --SPMR -B”.

#### ChIP-seq

H3K27ac ChIP-seq was performed as previously described^79^ on 20 million fresh primary ALL cells. Briefly, cells were crosslinked using 1% formaldehyde (diluted from sigma F87750) at room temp for 10 min. Crosslinking was stopped with 2.5M glycine (final concentration 0.125M). 5μg anti-H3K27Ac antibody (H3K27ac Rabbit pAb, Active Motif #39133) was bound to 200ul of protein G dynabeads (Invitrogen 10003D) overnight in 0.5% BSA/PBS. 20M fixed cells were lysed in 1mL Farnham lysis buffer (5mM PIPES pH 8, 85mM KCl, 0.5% NP40, 1x protease inhibitors (Roche 11836170001)) and passed through an 18G needle 10x. Nuclei were resuspended in 275ul of RIPA buffer (1x PBS, 1% NP40, 0.5% Sodium Deoxycholate, 0.1% SDS, 1x protease inhibitors) and sonicated using a Diagenode Bioruptor Plus on high power in 1.5mL tubes for 25 cycles (30s on/30s off). 5% input samples were taken, and the remaining sonicated chromatin was rotated with the antibody/protein G beads overnight at 4C. The next morning the beads were washed 5x with ice-cold LiCl buffer (100mM Tris pH 7.5, 500mM LiCL, 1% NP40, 1% sodium deoxycholate) and 1x with ice-cold TE buffer (10mM Tris pH 7.5, 1mM EDTA). DNA was then eluted from the beads using elution buffer (1% SDS, 0.1 M NaHCO_3_) at 65°C, vortexing 4x over 1 h. The eluted DNA and input DNA samples were then incubated at 65°C overnight to reverse crosslinks. DNA was purified using the QIAquick PCR purification kit (Qiagen 28104). DNA quantification was performed using the PicoGreen assay (Molecular Probes, Eugene, OR, P-7581). Sequencing libraries were generated from ChIP and input DNA by using the KAPA Hyper Prep kit (Roche, Basel, Switzerland, # 7962363001) according to the included manufacturer’s specifications, and quality was determined by using the Agilent TapeStation with D1000 screentape.

Amplified DNA libraries were sequenced on a Nova-seq 6000 or NovaSeq X+ (Illumina) using 150bp single-end sequencing. Adapter trimming was performed using TrimGalore (v0.6.6) with command options “--fastqc”. Read mapping was performed using Bowtie2 (v2.2.9)^64^ and hg19 genome index with custom command options “-X 2000 -S”. Read quality filtering was performed with Samtools with the “view” command and options “-q 20 -b”. Sorting was performed with Picard (v1.141) using the “SortSam” command and options “SORT_ORDER = coordinate”. Mitochondrial reads were removed using Samtools “view” command combined with command line filtering, “samtools view -h bam | awk '{if($3 ! = "chrM"){print $0}}' | samtools view -b - > bam”. Peak calling was performed with MACS2^67^ (v2.1.1) using command and options “macs2 callpeak -t bam -f BAM -g hs --nomodel --extsize 200 --SPMR -B”.

#### CUT&RUN

CUT&RUN was performed using the Epicypher Cutana CUT&RUN kit v3.0 (14–1048) according to the manufacturer’s instructions. Briefly, 500k cells were bound to ConA beads at room temperature in wash buffer for 10 min. Bead-bound cells were suspended in antibody binding buffer containing 0.01% digitonin and incubated overnight at 4°C on Nutator (Fisher Scientific S06622) 0.5 μg of each respective antibody (See key resources table). The next morning bead bound cells were washed twice with cell permeabilization buffer containing 0.01% digitonin to remove excess/unbound antibody. pAG-MNase was then added and allowed to bind the primary antibody for 10 min at room temperature. Immediately after binding, bead bound cells were washed twice with cell permeabilization buffer to remove excess/unbound pAG-MNase. Targeted chromatin digestion was started with the addition of 1 μL 100 mM calcium chloride and incubated for 2 h at 4°C. The digestion was stopped with the addition of stop buffer containing 0.5 ng per sample of E. Coli spike-in. Fragments were then released for 10 min at 37°C. The supernatant was then removed and subjected to DNA purification using the columns and buffers included in the kit. CUT&RUN DNA was quantified using the Quant-iT PicoGreen ds DNA assay (ThermoFisher). Libraries were prepared with HyperPrep Library Preparation Kit (Roche PN 07962363001) with modified PCR conditions:

Step 1 98C for 45s.

Step 2 98C for 15s.

Step 3 60C for 10s.

Step 4 72C for 1min.

Repeat steps 2–4. 13 cycles for input >5 ng, 15 cycles for input <5 ng)

Step 4 72C for 1min.

Libraries were analyzed for insert size distribution using the 2100 BioAnalyzer High Sensitivity kit (Agilent), 4200 TapeStation D1000 ScreenTape assay (Agilent), or 5300 Fragment Analyzer NGS fragment kit (Agilent). Libraries were quantified using the Quant-iT PicoGreen ds DNA assay (ThermoFisher) or by low pass sequencing with a MiSeq nano kit (Illumina). Paired-end sequencing was performed on a NovaSeq 6000 or NovaSeq X+ (Illumina). Data analysis of CUT&RUN samples was performed using Nextflow (2.10.6) and nextflow-core cutandrun pipeline (2.0.0) 74.

#### Promoter capture Hi-C

Arima promoter capture Hi-C (Arima product #s: A510008, A303010, A302010) was performed on B-ALL cell lines (697, Nalm6, RS411, REH, SUPB15, BALL1, SEM) or B-ALL patient samples (n = 10; BCR-ABL1, ETV6-RUNX1, KMT2A-rearranged, Ph-like, TCF3-PBX1 and B-other subytpes) according to the manufacturers provided instructions using unspecified proprietary buffers, solutions, enzymes, and reagents. Briefly, 10 million ALL cells were harvested, suspended in 5mL RT PBS which was brought to 2% formaldehyde by adding 37% methanol-stabilized paraformaldehyde for a 10-min fixation. For patient samples 1.5 to 5 million cells were fixed in 1% formaldehyde for 10 min. The amount of fixed cell suspension equal to 5μg of cell DNA was used for HiC. Cells were lysed with Lysis Buffer and conditioned with Conditioning Solution before their DNA was digested in a cocktail consisting of Buffer A, Enzyme 1, and Enzyme 2. The digested, fixed chromatin was biotinylated using Buffer B and Enzyme B before being ligated using Buffer C and Enzyme C. The fixed, biotinylated, ligated DNA was then subjected to reversal of crosslinking and digestion of proteins before being purified. 100ul containing 1500ug of purified large proximally ligated DNA was fragmented for 24 cycles (30s on/30s off) using a Diagenode Bioruptor Plus bath sonicator. The fragmented DNA was then subjected to two-sided size selection targeting fragments between 200 and 600bp using AMPure XP DNA purification beads. Size selected DNA was then subjected to biotin enrichment using T1 streptavidin beads. Bead bound, enriched HiC DNA was then subjected to Arima library prep. Briefly, the sample underwent end repair followed by adapter ligation, at which point the sample was then subjected to 10 cycles of PCR amplification. The library DNA was then purified using AMPure XP DNA purification beads. The HiC library was then subjected to Arima promoter capture enrichment. The library was precleared of biotinylated DNA using T1 streptavidin beads before being subjected to promoter enrichment with biotinylated RNA probes. After washing, the captured fragments were then amplified an additional 13 PCR cycles.

Amplified DNA libraries were sequenced on a Nova-seq 6000 or NovaSeq X+ (Illumina) using 150bp paired-end sequencing. Analysis of promoter capture HiC data was performed using the Arima CHiC pipeline (v1.4, https://github.com/ArimaGenomics/CHiC). Briefly, this pipeline uses HiCUP v0.8.0^72^ for mapping and quality assessment of promoter capture HiC data and CHiCAGO^80^ to identify significant looping interactions in the promoter capture HiC data using 3kb resolution and adjusted p value <0.05. Files were processed at 3kb resolution with command “bash Arima-CHiC-v1.4.sh” with key custom options including: “-W 1 -Y 1 -Z 1 -P 1 -day Digest_hg19_Arima.txt -b human_GW_PC_S3207364_S3207414_hg19.uniq.bed -R hg19_chicago_input_3kb.rmap -B hg19_chicago_input_3kb.baitmap -O hg19”. The following input files: hiccup genome digest (Digest_hg19_Arima.txt), probe design file (human_GW_PC_S3207364_S3207414_hg19.uniq.bed), rmap file (hg19_chicago_input_3kb.rmap), baitmap file (hg19_chicago_input_3kb.baitmap) and corresponding hg19 3kb resolution CHiCAGO design files (∗.npb, ∗.poe, ∗.nbpb) were sourced from Arima FTP server (ftp://ftp-arimagenomics.sdsc.edu/pub/ARIMA_Capture_HiC_Settings/). All significant intrachromosomal chromatin interactions spanning less than 2Mb were concatenated from all B-ALL cell lines to create a comprehensive library of pan-B-ALL cell line promoter capture chromatin loops. Genomic regions representing separate loop ends were compiled to facilitate overlap determinations with B-ALL patient chromatin accessible regions of interest using “bedtools intersect”.

#### Functional genomic data

Transcriptomic and SNP genotyping data from B-ALL patient samples were obtained from St. Jude Children’s Research Hospital. Normal B cell ATAC-seq^10^^,^^25^ were downloaded from NCBI (GSE122989 and GSE74912). B-ALL cell line ATAC-seq and H3K27ac tracks associated with dCas9-KRAB repressor targeting or CRISPR/Cas9 deletions and investigation were downloaded from GEO under accession numbers GSE186942 ^27^ and GSE129066.^29^ B-ALL cell histone modification ChIP-seq datasets (H3K27ac, H3K4me1 and H3K27me3) were downloaded from the Blueprint Epigenome Consortium (https://www.blueprint-epigenome.eu/). B-ALL ATAC-seq data in 24 patient samples used for validation of our classification model were downloaded from GEO under the accession number GSE161501.^29^ Expression quantitative trait loci (eQTL) data was obtained from previous studies.^29^

#### ATAC-seq regions of interest selection

Accessible chromatin sites analyzed throughout this work were selected using a reproducible ATAC-seq peak summit approach as follows. Peak summits were generated for each subtype on a subtype-merged basis and narrowPeak regions for each individual patient sample. Any subtype-merged peak summit not reproducible among multiple individual patient sample narrowPeaks was excluded for analysis. All reproducible, subtype-merged summits were then extended upstream and downstream to an interval size of 301bp and merged if overlapping. Finally, any interval overlapping hg19 blacklist regions were eliminated yielding the final set of ATAC-seq regions of interest used in further analysis. The ChIPseeker^56^ R-package was used for genomic annotation of all genomic intervals throughout this work. Three B-ALL subtype patient samples (IKZF1 N159Y, iAMP21 and *ETV6*::*RUNX1-Like*) were included in B-ALL versus Pro-B cell analyses but were excluded from additional studies due to limited sample size.

#### B cell progenitor versus B-ALL cell comparisons

All samples were required to pass an ATAC-seq quality score cutoff to be included in analysis. ATAC-seq quality scores were determined by calculating ATAC-seq read enrichment around transcription start sites. Normal progenitor cells were sourced from NCBI GEO (GSE122989 and GSE74912). In total, 185,135 merged peak intervals from progenitors were used as starting input across the differentiating cell types. These merged peaks were subsequently windowed into 250bp intervals (520,095) to better detect subtle differences between progenitors. DESeq2^57^ using the Wald statistical test across these 520,095 250bp regions was utilized to calculate differential chromatin accessibility between B cell progenitors cells comparing a single progenitor to all other progenitors as a collective group. 42,344 out of 520,095 genomic intervals were identified (p-adjusted filter of <0.005 and an absolute value log2(fold change) ≥ 1) as distinctive for B cell progenitors, approximately 8% of the total.^57^ Heatmap of hierarchical clustering between B cell progenitor cells and B-ALL samples were generated using the pheatmap R-package and variance stabilized ATAC-seq signal from DESeq2. DESeq2^57^ using the Wald statistical test on the merged set of 217,240 B-ALL accessible chromatin sites was utilized to calculate differential chromatin accessibility between normal Pro-B cells and B-ALL patient samples. Differential chromatin accessibility between normal Pro-B cells and B-ALL patient samples was defined as chromatin regions passing a p-adjusted filter of <0.05 and an absolute value log2(fold change) ≥ 1. A variance stabilized transform function within DESeq2 was applied to the ATAC-seq read counts matrix and *Z* score signal across all samples at Pro-B cell and B-ALL enriched DASs was used for hierarchical clustering using the pheatmap R-package.^58^ Code for heatmap generated between Pro-B and B-ALL cells used: pheatmap(ProB_vs_BALL_results_vst_prog_ALL, color = plasma(11), cellwidth = 3, cellheight = NA, cluster_rows = T, cluster_cols = F, scale = "none", show_rownames = FALSE, show_colnames = FALSE, annotation_col = prog_BALL_anno_df2, annotation_colors = ann_colors2, gaps_col = c(3)). The Genomic Regions Enrichment of Annotations Tool (GREAT)^78^ was used to identify candidate target gene sets and ontologies associated with DASs. TOBIAS^12^ was used to identify TF footprints at accessible chromatin sites.

#### Subtype-enriched chromatin accessibility analysis

DESeq2^57^ using the Wald statistical test was utilized to calculate differential chromatin accessibility among B-ALL subtypes. Cohorts representing a single subtype were compared to all other B-ALL patient samples not belonging to the single subtype as a collective group. This pairwise comparison was completed for all subtypes and samples with sufficient sample numbers (N > 1). Subtype-enriched chromatin accessible regions were required to pass filters of p-adjusted <0.05 and an absolute value log2(fold change) ≥ 1. Subtype-enriched regions were additionally required to be exclusively differential in a single subtype, regions appearing as differential in multiple subtypes were excluded. A variance stabilized transform function within DESeq2 was applied to the ATAC-seq read counts matrix specific to subtype-enriched loci prior to visualization with the pheatmap R-package. TOBIAS^12^ was used to identify TF footprints at accessible chromatin sites. The Principal Component Analysis-Linear Discriminant Analysis (PCA-LDA) subtype classification model was constructed stepwise by first PCA transformation of subtype-enriched ATAC-seq counts, then applying LDA on an optimized number of principal components.

#### Transcription factor-target gene network analysis

Enhancer and target gene prediction for network construction was analyzed with the ABC enhancer algorithm (https://github.com/broadinstitute/ABC-Enhancer-Gene-Prediction) 26. In brief, inputs for the ABC enhancer algorithm included, B-ALL enriched DASs, merged B-ALL patient ATAC-seq, H3K27Ac ChIP-seq, Arima promoter capture Hi-C contact counts with ABC score threshold at 0.04.

#### ATAC-QTL identification

VCF (Variant Call Frequency) files were sourced from St. Jude Children’s Research Hospital genotyping. Variants in this dataset represented a mixture of both directly genotyped and imputed variants. Imputation was performed via the Michigan imputation server (version 1.6.5) using minimac4 for imputation, eagle-2.4 for phasing and the TOPMed reference panel. The final variant list for analysis with RASQUAL^30^ was restricted to variants within B-ALL open chromatin regions yielding 914,406 variant SNPs in total. Allele specific ATAC-seq read counting for open chromatin region SNPs was performed with the RASQUAL supplied helper script which utilizes the GATK ASEReadCounter tool. All SNPs were required to have an imputation quality R^2^ of ≥ 0.80 for final inclusion after running RASQUAL. Significant ATAC-QTLs for each region were identified with a genome-wide computed FDR of 10%.

#### CRISPR-Cas9 deletion of *cis*-regulatory elements

Targeted deletion pools were generated using CRISPR-Cas9 technology. Briefly, 500,000 parental cells from the corresponding cell line (Nalm6, 697 or SUPB15) were transiently transfected with two precomplexed ribonuclear protein (RNPs) consisting of 75 pmol of each chemically modified sgRNA (Synthego) and 60 pmol of 3X NLS *Sp*Cas9 protein (St. Jude Protein Production Core) via nucleofection (Lonza, 4D-Nucleofector X-unit) using solution P3 and program CV-104 for Nalm6, CA-137 for 697, CM-138 for SUPB15 cells in a small (20μl) cuvette according to the manufacturer’s recommended protocol (see Table S10). Three days post nucleofection, cell pellets of approximately 10,000 cells were lysed and used to generate deletion specific amplicons that were run on a 1% agarose gel and sequenced via targeted next generation sequencing as previously described.^81^ Final pools were authenticated using the PowerPlex Fusion System (Promega) performed at the Hartwell Center (St. Jude) and tested negative for mycoplasma by the MycoAlertPlus Mycoplasma Detection Kit (Lonza). Editing construct sequences and relevant primers are listed in the table below. qPCR on cDNA from deleted and wild-type parental cells was performed using TaqMan probes for *SLC2A9* (Hs01119178_m1, ThermoFisher), *CDK14* (Hs00953416_m1, ThermoFisher) and *SH3BP5L* (Hs00944382_m1, ThermoFisher).

For gene expression measurements, parental (WT) and *cis*-regulatory element-deleted cells (Del) were cultured in RPMI 1640 media (supplemented with 10% FBS, 1% Penicillin/Streptomycin, and 1% L-Glutamine). Cells were collected and lysed with RLT/BME mixture (1000 μL:10 μL) and processed for total RNA extraction (Qiagen #74104). cDNA synthesis was done using the High-Capacity RNA-to-cDNA kit (Applied Biosystems #4387406). TaqMan Fast Advanced Master Mix (Applied Biosystems #4444557) and TaqMan Gene Expression Assays probe (Thermo) were used to prepare RT-PCR reactions. Taqman probes used include: Hs00944382_m1 (*SH3BP5L*), Hs01119178_m1 (*SLC2A9*), Hs00953418_m1 (*CDK14*) and Hs00427620_m1 (*TBP*, endogenous control). The recommended Taqman Fast Advanced Master Mix PCR conditions were used to run the samples and the samples were run on a QuantStudio3 Real-Time PCR system.

For cell proliferation analyses, parental (WT) and *cis*-regulatory element-deleted cells (Del) were cultured in RPMI 1640 media (supplemented with 10% FBS, 1% Penicillin/Streptomycin, and 1% L-Glutamine) and plated on a 96 well plate using 0.2 × 10ˆ5 cells per well (n = 3 per group). Cells proliferation was measured for up to 17 days by adding fresh media and expanding cells when confluence was reached. Absolute cell count was done by Trypan blue method using TC20 automated cell counter (Bio-Rad, #1450102) at different time points.

#### CRISPRi dCas9-KRAB enhancer targeting

Putative subtype-specific enhancer regions were selected based DESeq2 output parameters including: log2FoldChange >2, baseMean >10, a lfcSE <20% of the log2FoldChange value, and autosomal chromosome location.

To design sgRNAs targeting enhancers, we used FlashFry^55^ to identify and score all candidate sgRNAs in a 2-kb window centered on ATAC-Seq peaks of interest. Candidates were kept that met the following scoring criteria: Doench2014OnTarget > 0.1, Hsu2013 > 50, JostCRISPRi_specificityscore > 0.1, dangerous_GC = = “NONE,” dangerous_polyT = = “NONE,” dangerous_in_genome = = “IN_GENOME = 1”, otCount <500. The final sgRNAs used for experiments were selected on the bases of shortest distance to ATAC and highest on-target score. Additional, previously described^27^ non-targeting (non-complementary to human genome) and non-coding (region of the human genome without regulatory relevance) sgRNA were selected as negative controls. 2 sgRNAs were selected for each putative enhancer (see Table S11). Complementary oligonucleotides encoding sgRNA sequences plus appropriate overhangs were synthesized (IDT), annealed, and cloned into BsmBI-digested sgOpti (Addgene #85681).

CRISPRi-ready SEM and Nalm6 cell populations with dox-inducible dCas9-KRAB and a GFP reporter (i.e., CiG) were generated as follows. Cells were transduced with lentivirus produced from TRE3-KRABdCas9-IRES-GFP and pLVX-EF1alpha-Tet3G vectors. Cells were serially sorted for GFP+ cells after doxycycline induction, for GFP-negative cells without doxycycline induction, and again for GFP+ cells after doxycycline induction.

For enhancer-targeting sgRNA experiments, SEM-CiG and NALM6-CiG cells were transduced with control and repeat enhancer-targeting sgRNA lentivirus by spinfection (see Table S11 for sgRNA sequences). Cells were treated 48 h after transduction with 1 μg/mL puromycin and 100 ng/mL doxycycline for an additional 72 h and were harvested (5 days after transduction) for RNA extraction and RT-qPCR. RT-qPCR primers against target genes in proximity to proposed subtype-specific enhancers were designed by Primer-Blast^70^ with following settings modifications: GC clamp = 1, Exon Junction Span = “Primer must span an exon-exon junction”. Gene targets were selected by proximity to putative enhancers and evidence of promoter-enhancer linkage. For each sgRNA treatment, 2 biological replicates and 3 RT-qPCR technical replicates per biological replicates were generated. Undetermined (undetected) values for technical replicates were imputed utilizing the ‘qpcrImpute’ method from the R package ‘nondetects’.^69^ RT-qPCR data were analyzed by the delta-delta Ct (2–ΔΔCt) method, averaging technical replicates. Biological replicate gene expression changes were pooled into negative control and enhancer knockdown groups (n = 2 sgRNAs x 2 biological replicates = 4) for determination of significance of gene knockdown by two-sample t test with unequal variance.

#### Luciferase reporter assays

A 301-bp fragment of DNA sequence centered on reference or the alternative alleles of rs17481869 (see Table S12) was cloned upstream of the minimal promoter into the pGL4.23 vector (Promega, E841A). Ten million Nalm6, 697 or SUPB15 cell line were co-transfected with test DNA sequence-cloned pGL4.23 luciferase and renilla plasmid constructs using the Neon transfection system (Thermo Fisher Scientific, MPK5000) with cell line optimized transfection parameters (Nalm6 = 1600V, 20ms, 1p; 697 = 1600V, 10ms, 3p; SUPB15 = 1450V, 20ms, 2p). After 24 h, firefly luciferase and renilla activity was measured on a BioTek Cytation1 plate reader (Agilent) using the Dual Luciferase Reporter Assay System (Promega, E1960). Luciferase activity was calculated as the ratio of firefly luciferase to Renilla luciferase activity.

#### Design of graphical abstract

The graphical abstract for this article was created with BioRender.com under a subscription plan for the Department of Pharmacy and Pharmaceutical Sciences at St. Jude Children’s Research Hospital, that includes publishing rights for journals and additional academic purposes.

### Quantification and statistical analysis

Initial chromatin accessibility intervals for individual cell line and patient samples before merging were filtered using the MACS2 narrowPeak file reported Benjamini-Hochberg corrected p value (q-value) requiring a q-value <0.05. Differential chromatin accessibility among B cell progenitor defining genomic loci was determined with DESeq2 using the Wald statistical test (covariates: none) requiring a Benjamini-Hochberg corrected p value < 0.005 and log2FoldChange ≥ 1 or ≤1. Differential chromatin accessible regions were not required to be unique to a single progenitor comparison. Differential chromatin accessibility comparing B-ALL patient samples with normal Pro-B samples was determined with DESeq2 using the Wald statistical test (covariates: none) requiring a Benjamini-Hochberg corrected p value < 0.05 and log2FoldChange ≥ 1 or ≤1. Differential chromatin accessibility comparing singular B-ALL subtypes with all other B-ALL subtype samples was determined with DESeq2 using the Wald statistical test (covariates: TSS enrichment, hospital site of origin, and sequencing run) requiring a Benjamini-Hochberg corrected p value < 0.05 and log2FoldChange ≥ 1 or ≤1. Regions demonstrating subtype enriched or depleted chromatin accessibility were required to be singularly unique to that subtype and not observed in any other subtype comparison. Patient samples representing ALL subtypes with only a single sample were excluded from subtype focused chromatin accessibility analyses. Additional details of quantification and statistical analyses can be found in methods details, figure legends and the github repository.

### Additional resources

Additional data about patient samples utilized in this study may be requested through the St. Jude Cloud data portals: https://platform.stjude.cloud/. A subset of patient samples in this study are associated with clinical trial TOT17 (NCI-2017-00582).

## Supplemental information

Document S1. Figures S1–S11 and Tables S10–S12

Data S1. Differential TF footprint scores at subtype-enriched and subtype-depleted DAS, related to Figure 5

Table S1. List of B-ALL molecular subtype patient samples, related to STAR Methods

Table S2. Reproducible B-ALL accessible chromatin sites within B-ALL subtypes, related to Figure 1

Table S3. Chromatin-accessibility regions defining differences between B-progenitors, related to Figure 2

Table S4. B-ALL versus normal Pro-B-enriched DASs, related to Figure 3

Table S5. Select enriched terms for GREAT gene ontology analysis for B-ALL-enriched DASs, related to Figure 3

Table S6. Select enriched terms for GREAT gene ontology analysis at AP-1-associated B-ALL-enriched DASs, related to Figure 3

Table S7. List of subtype-enriched DASs, related to Figure 4

Table S8. List of ATAC-QTLs, related to Figure 7

Table S9. List of ATAC-QTLs that are eQTLs, related to Figure 7

Document S2. Article plus supplemental information
