# Supplementary material for: Epigenomic mapping reveals distinct B cell acute lymphoblastic leukemia chromatin architectures and regulators
Source: Cell Genom. 2023 Nov 20;3(12):100442. doi: 10.1016/j.xgen.2023.100442 (PMC10726428; doi:10.1016/j.xgen.2023.100442)
Supplement: Document S1. Figures S1–S11 and Tables S10–S12 [file mmc1.pdf]

Cell Genomics, Volume *3*

Supplemental information

Epigenomic mapping reveals distinct B cell acute lymphoblastic leukemia chromatin architectures and regulators

Kelly R. Barnett, Robert J. Mobley, Jonathan D. Diedrich, Brennan P. Bergeron, Kashi Raj Bhattarai, Alexander C. Monovich, Shilpa Narina, Wenjian Yang, Kristine R. Crews, Christopher S. Manring, Elias Jabbour, Elisabeth Paietta, Mark R. Litzow, Steven M. Kornblau, Wendy Stock, Hiroto Inaba, Sima Jeha, Ching-Hon Pui, Charles G. Mullighan, Mary V. Relling, Shondra M. Pruett-Miller, Russell J.H. Ryan, Jun J. Yang, William E. Evans, and Daniel Savic

Supplemental Information

Epigenomic mapping reveals distinct B-cell acute lymphoblastic leukemia chromatin architectures and regulators

Kelly R. Barnett, PhD, Robert J. Mobley, PhD, Jonathan D. Diedrich, PhD, Brennan P. Bergeron, PhD, Kashi Raj Bhattarai, PhD, Alexander C. Monovich, Shilpa Narina, Wenjian Yang, PhD, Kristine R. Crews, PharmD, Christopher S. Manring, MBA, Elias Jabbour, MD, Elisabeth Paietta, PhD, Mark R. Litzow, MD, Steven M. Kornblau, MD, Wendy Stock, MD, Hiroto Inaba, MD, PhD, Sima Jeha, MD, Ching-Hon Pui, MD, Charles G. Mullighan, MBBS (Hons), MSc, MD, Mary V. Relling, PharmD, Shondra M Pruett-Miller, PhD, Russell J.H. Ryan, Jun J. Yang, PhD, William E. Evans, PharmD and Daniel Savic, PhD

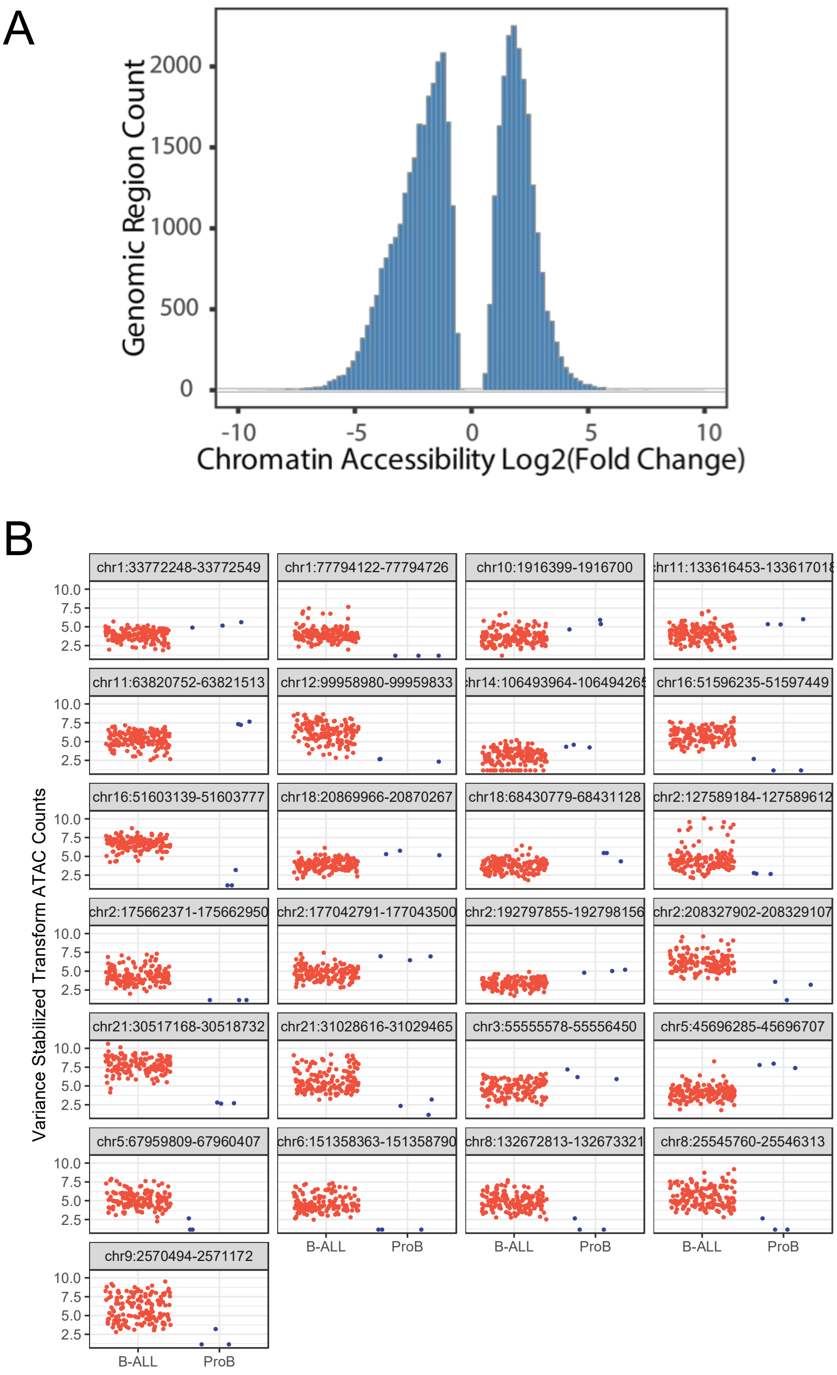

Figure S1: Analysis of differential accessibility between Pro-B cells and B-ALL, related to Figure 3. (A) Histogram of $\log_2$-adjusted fold change in ATAC-seq signal at significant DAS between Pro-B cells and B-ALL patient samples. **(B)** DESeq2 variance stabilized ATAC-seq counts for individual peaks between B-ALL patient samples (red, left) and ProB cell samples (blue, right).

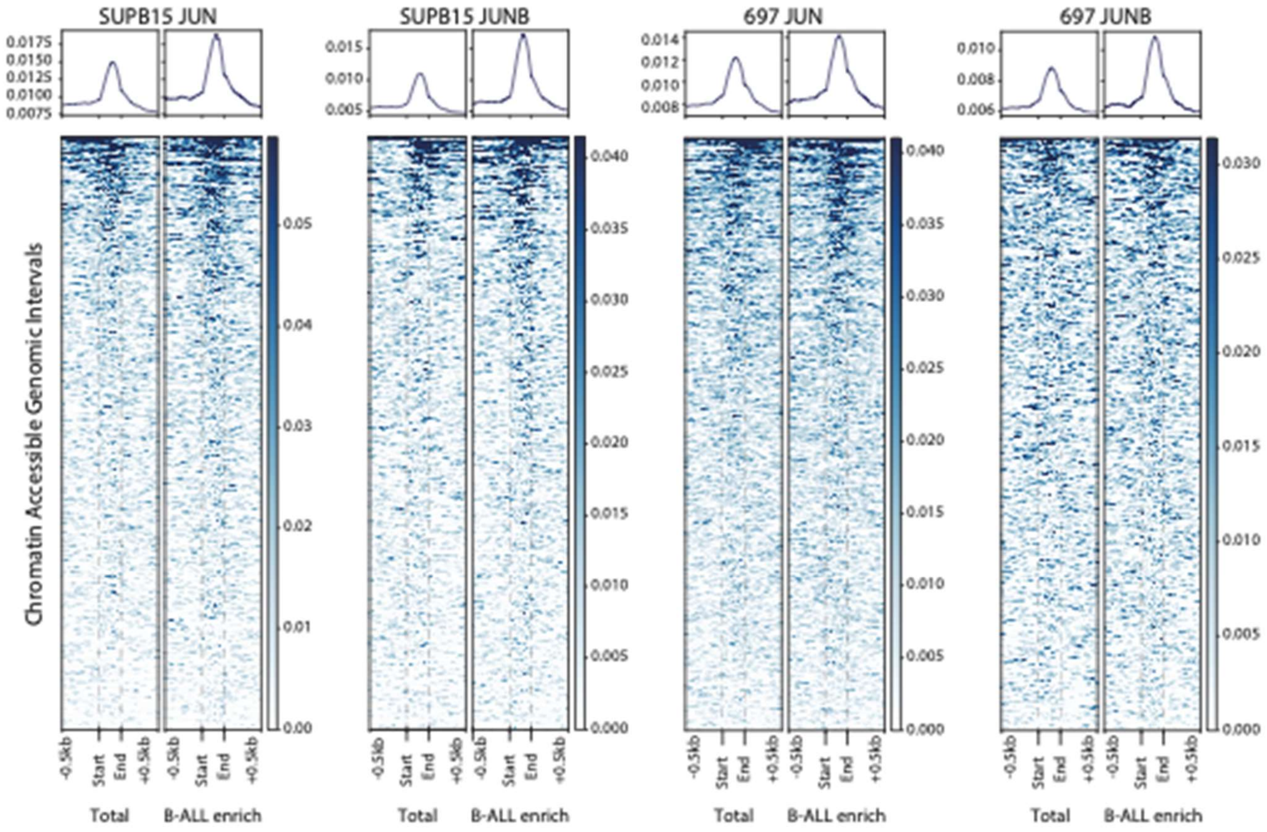

Figure S2: JUN and JUNB CUT and RUN read enrichment at B-ALL accessible chromatin sites, related to Figure 3. JUN and JUNB CUT and RUN enrichment heatmap at all B-ALL accessible chromatin sites (Total, N=217,240 regions; left) and B-ALL enriched DAS (B-ALL enrich, N = 23,372; right) in SUPB15 and 697 cells is shown. Dotted lines define chromatin accessible peak intervals. Genomic intervals +/- 5 kilobases from peaks are shown for context. Rows in adjacent pairs of heatmaps are unaligned.

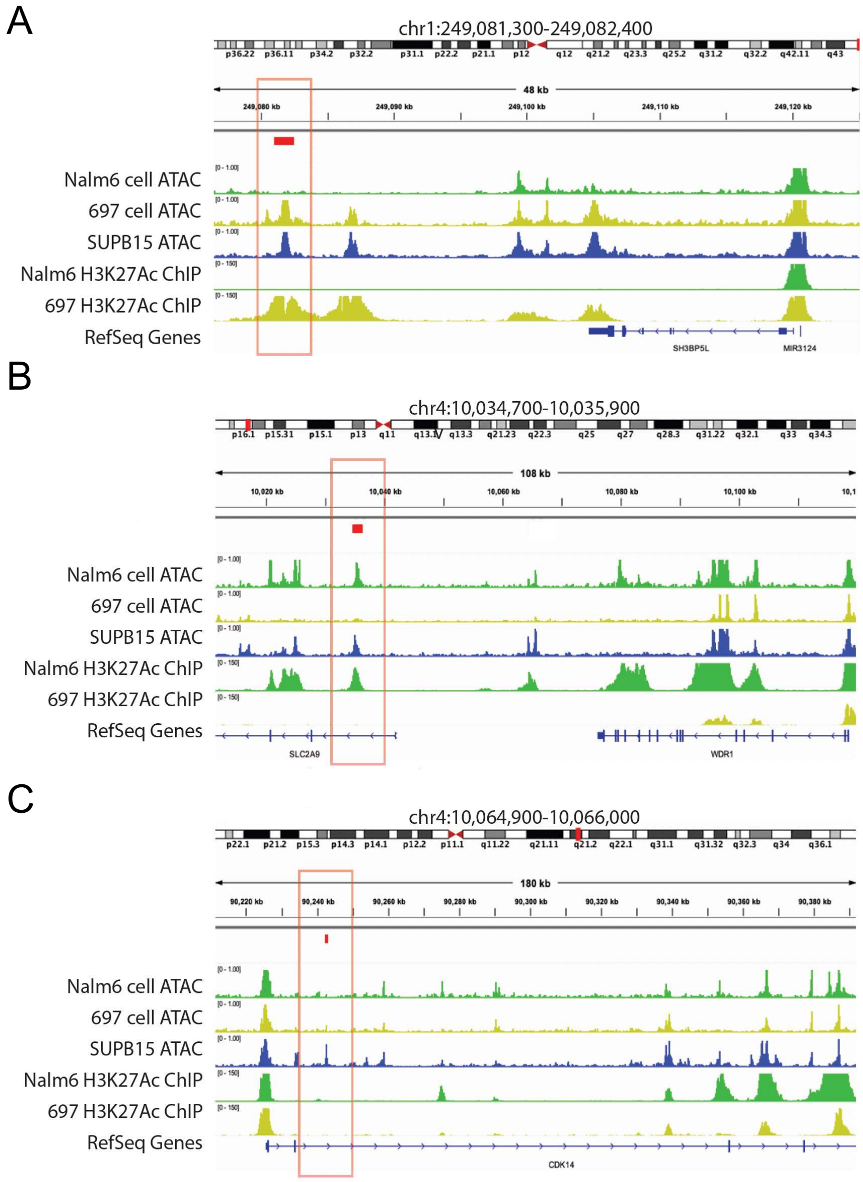

Figure S3: Genome browser images of *cis*-regulatory elements targeted for deletion using CRISPR-Cas9 genome editing, related to Figure 3. Genome browser views of B-ALL cell line ATAC-seq and H3K27Ac ChIP-seq across loci targeted for CRISPR-cas9 mediated genomic deletion of B-ALL *cis*-regulatory elements. Coordinates (hg19) for each deletion are provided above and the genomic locations (red rectangles and outlined in red) are provided in each browser image for *cis*-regulatory element deletions near *SH3BP5L* **(A)**, *SLC2A9* **(B)** and *CDK14* **(C)**.

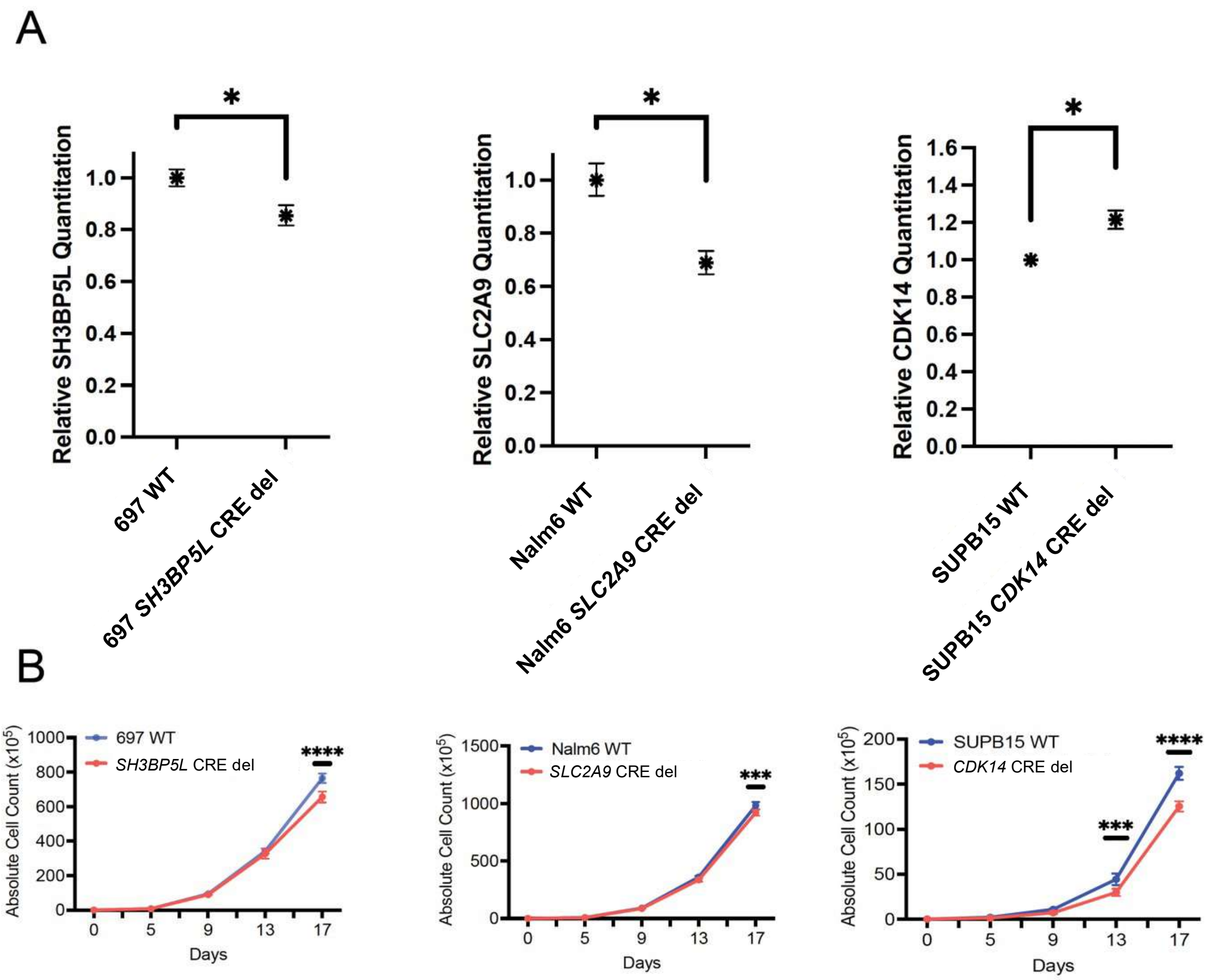

Figure S4: ***Cis*-regulatory element deletion effects on gene expression and cell proliferation, related to Figure 3. (A)** RT-qPCR analysis of nearby gene expression within heterogeneous CRISPR-Cas9 deletion cell pools targeting putative B-ALL *cis*-regulatory elements (n=3 per sample). **(B)** Cell proliferation analysis of WT B-ALL cells or B-ALL cells deleted for putative B-ALL *cis*-regulatory elements (n=3 per sample). Experiments were performed in 697 (*SH3BP5L*), Nalm6 (*SLC2A9*) and SUPB15 (*CDK14*) cells.

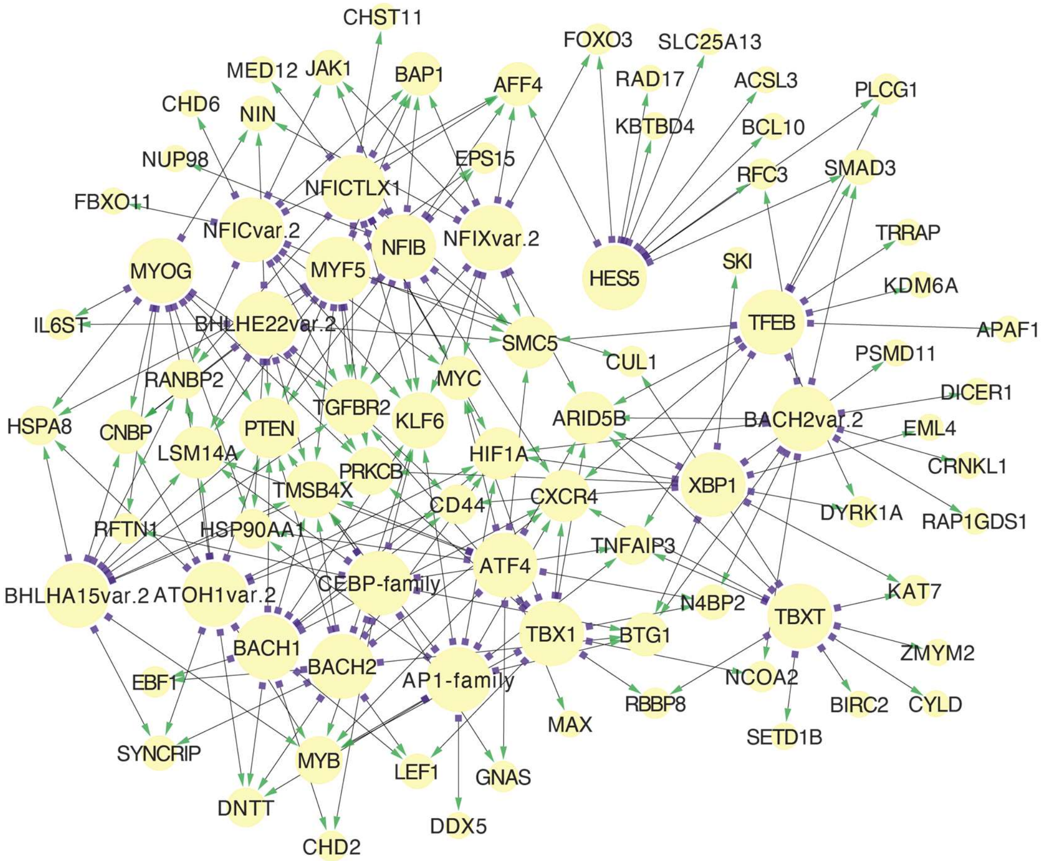

Figure S5: TF-target gene relationships using B-ALL-enriched DAS TF footprinting and B-ALL cell line promoter capture Hi-C, related to Figure 3. Transcription factor and target gene network of DAS with higher accessibility in B-ALL (B-ALL-enriched). Network is subset for top transcription factor footprints across DAS ranked by the top mean log_2-adjusted fold change transcription factor footprint signal. Target genes determined with B-ALL cell line promoter capture Hi-C are subset for a cancer implicated gene set ranked by the top expressed genes. Network connections are colored as transcription factors (purple blocks) to target gene (green arrow heads) pairs. Select expansive and highly similar transcription factor motif families are grouped (AP-1 and CEBP; AP1-family and CEBP-family).

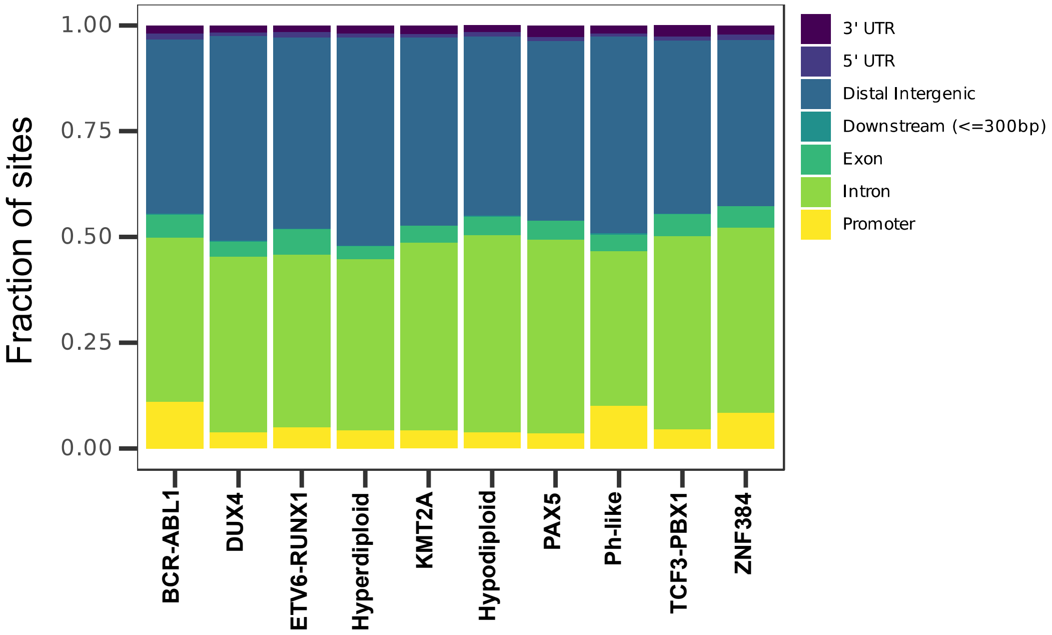

Figure S6: Genomic distributions of subtype-enriched DAS, related to Figure 4. Genomic annotations of subtype-enriched DAS for each B-ALL subtype.

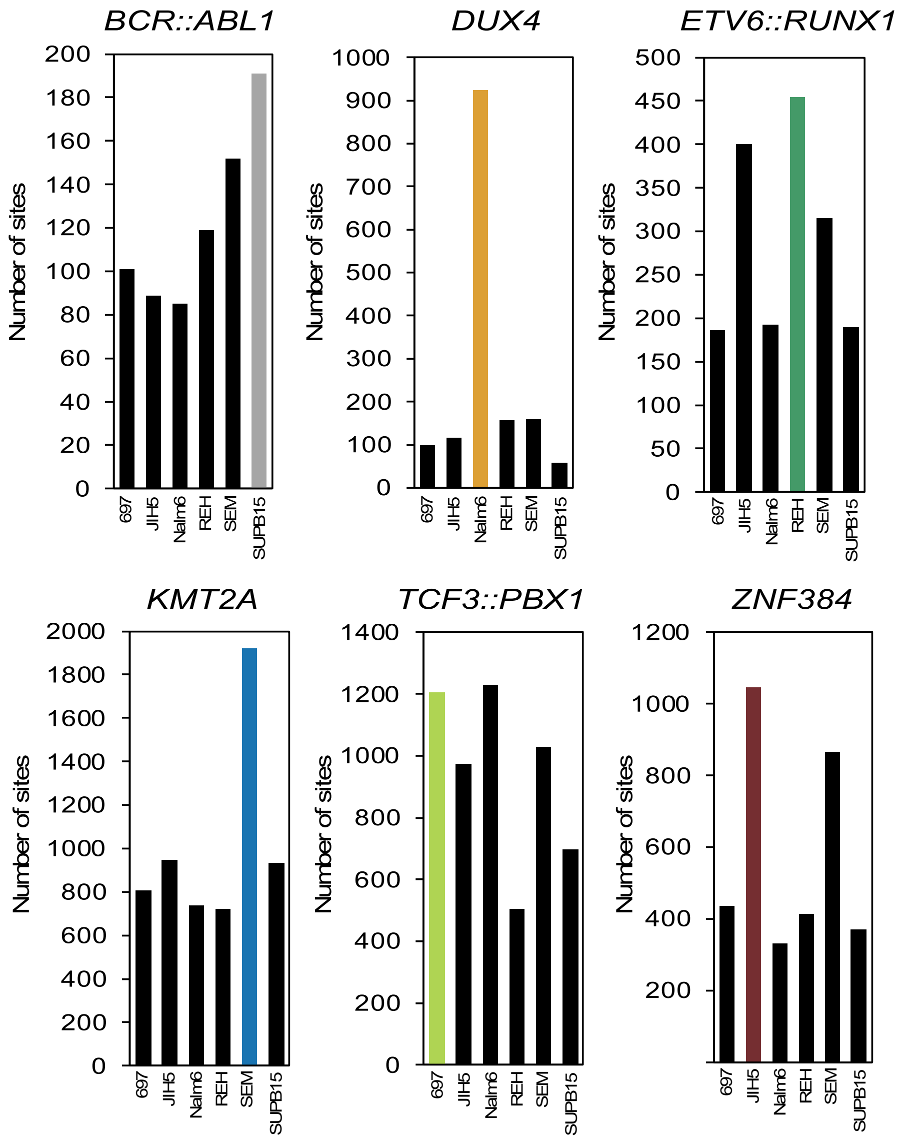

Figure S7: Comparisons of subtype-enriched DAS across diverse B-ALL cell lines, related to Figure 4. Across a panel of B-ALL cell lines (697, JIH5, Nalm6, REH, SEM and SUP-B15; x-axis), bar plots delineate the number of (from left to right) *BCR::ABL1*, *DUX4*-rearranged (DUX4), *ETV6::RUNX1*, *KMT2A*-rearranged (KMT2A), *TCF3::PBX1* and *ZNF384*-rearranged (ZNF384) DAS that exhibit the strongest accessibility in each B-ALL cell line. Color denotes the corresponding B-ALL cell line for each subtype-enriched DAS and black denotes B-ALL cell lines from opposing subtypes.

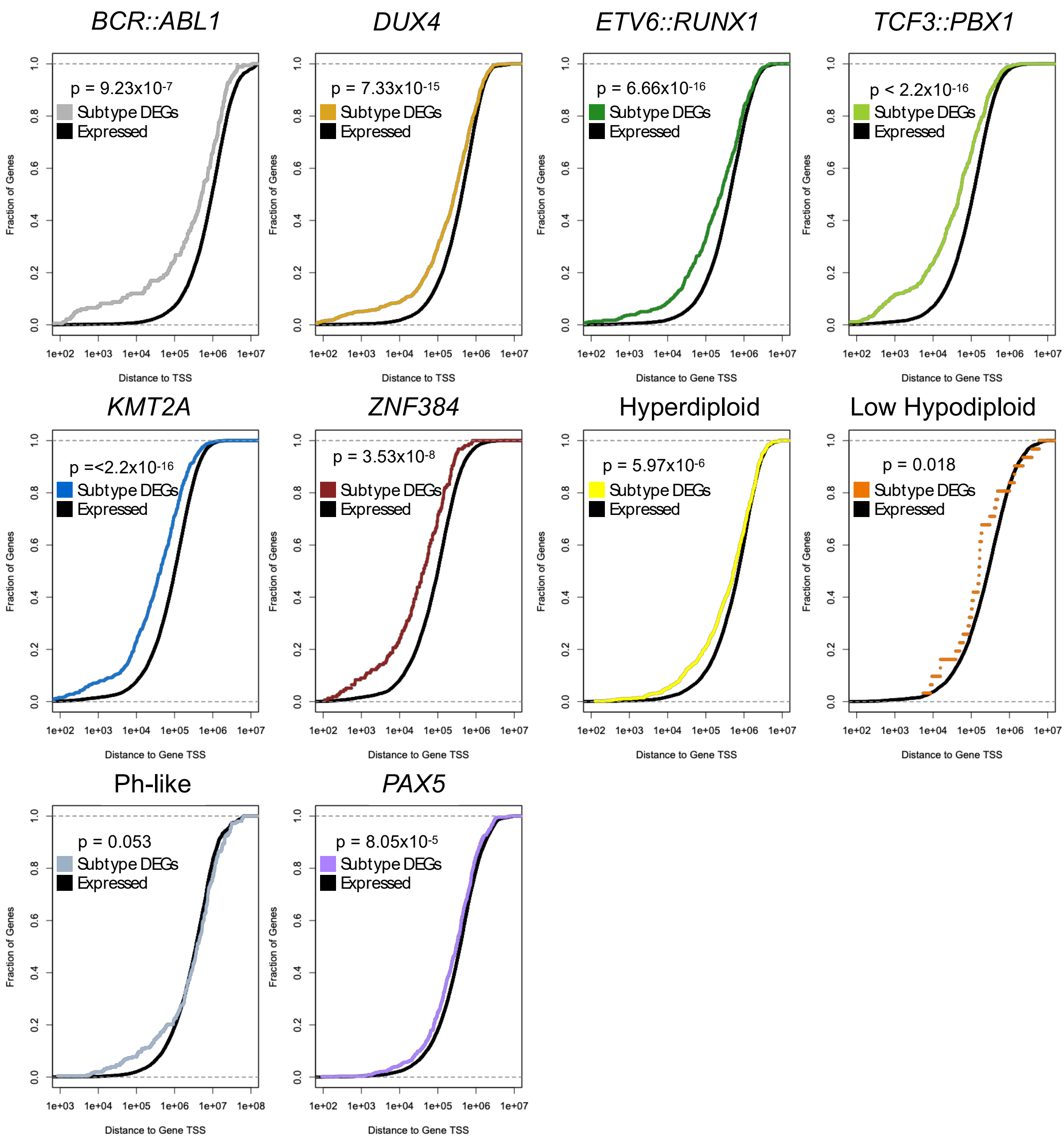

Figure S8: Distance comparisons of subtype-enriched DAS with DEGs uniquely up-regulated in each subtype, related to Figure 4. Cumulative distribution function comparing the fraction (y-axis) of subtype up-regulated genes (Subtype DEGs; red) and all expressed subtype gene (Expressed genes; black) at different distance cutoffs from subtype-enriched DAS and their transcription start sites (x-axis). Kolmogorov-Smirnov (K-S) p-values are provided for each B-ALL subtype.

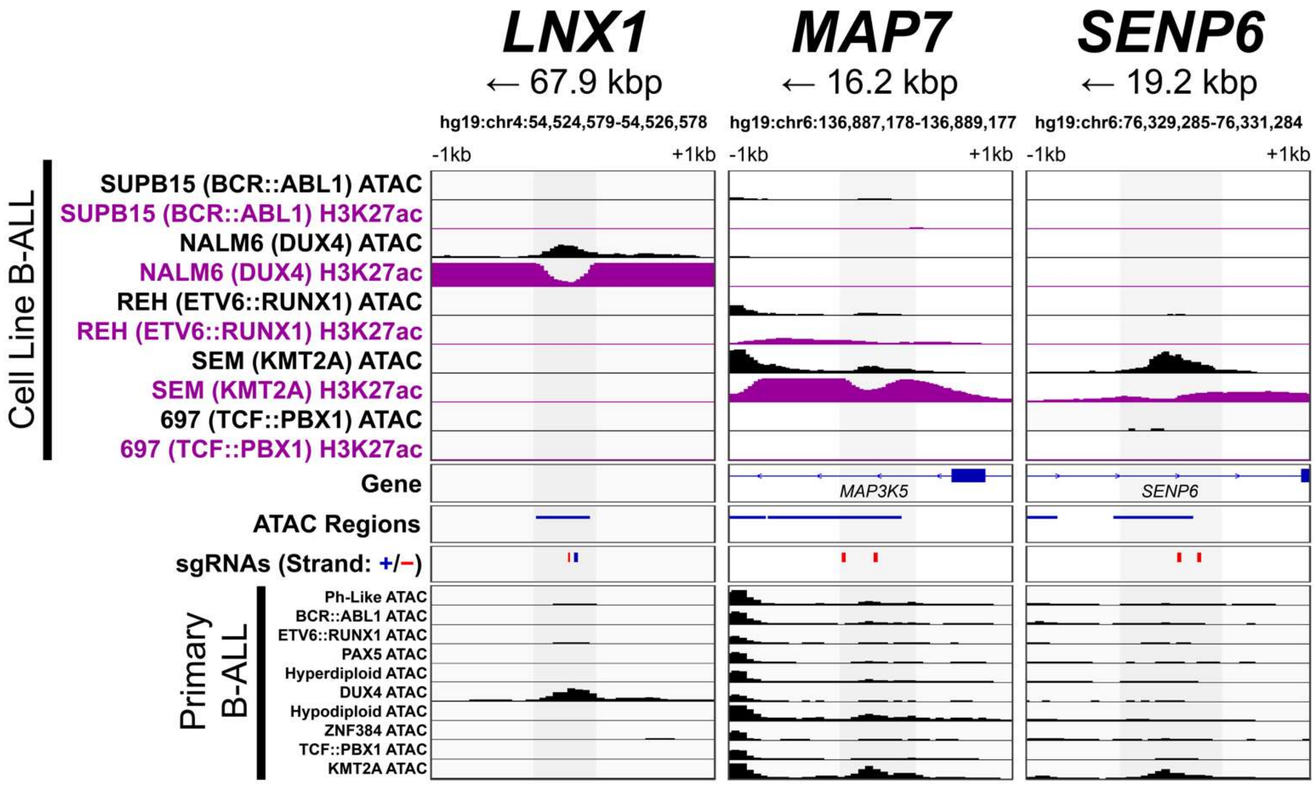

Figure S9: Genome browser images of *cis*-regulatory elements targeted by CRISPRi, related to Figure 4. Detail (2,000 bp window) of putative enhancer *cis*-regulatory element regions targeted with dCas9-KRAB CRISPRi. Cell line ATAC-seq and H3K27ac ChIP-seq for representative enhancers with subtype-specific enrichment. Distance and position with respect to target gene are listed at the top with associated B-ALL subtype in parentheses. Cell line H3K27ac ChIP-seq tracks are shown in purple (scale, 15 fragments per million, fpm) and ATAC-seq tracks in black (scale 7.5 fpm). Subtype ATAC-seq tracks merged from primary B-ALLs are shown in black (scale 2 fpm). The position of sgRNA sequences used in X. are shown (blue = positive strand, red = negative strand).

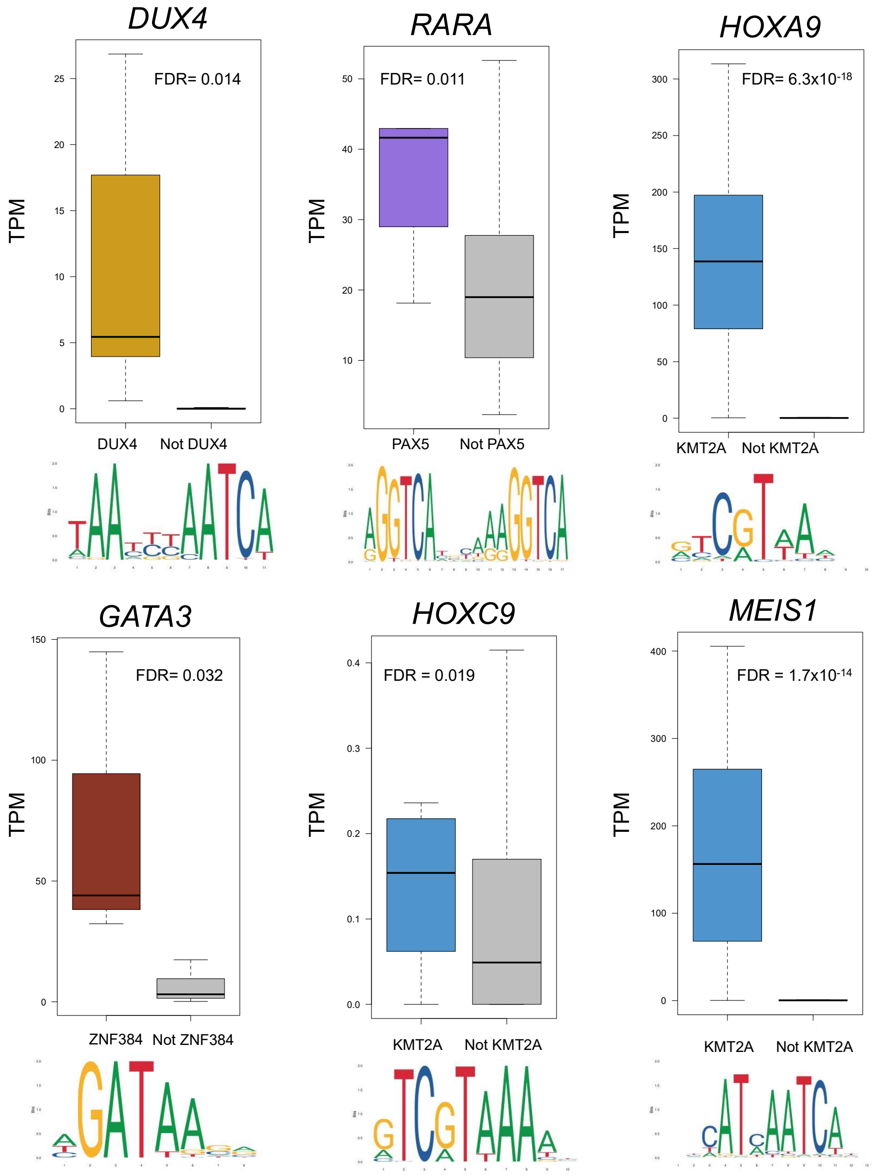

Figure S10: Gene expression of TFs harboring subtype-enriched footprints, related to Figure 5. RNA-seq transcripts per million (TPM) expression of key TFs with subtype-enriched footprints that are also up-regulated in the corresponding subtype (in red) versus all other subtypes (gray). DESeq2 differentially expressed gene FDR significance values are provided.

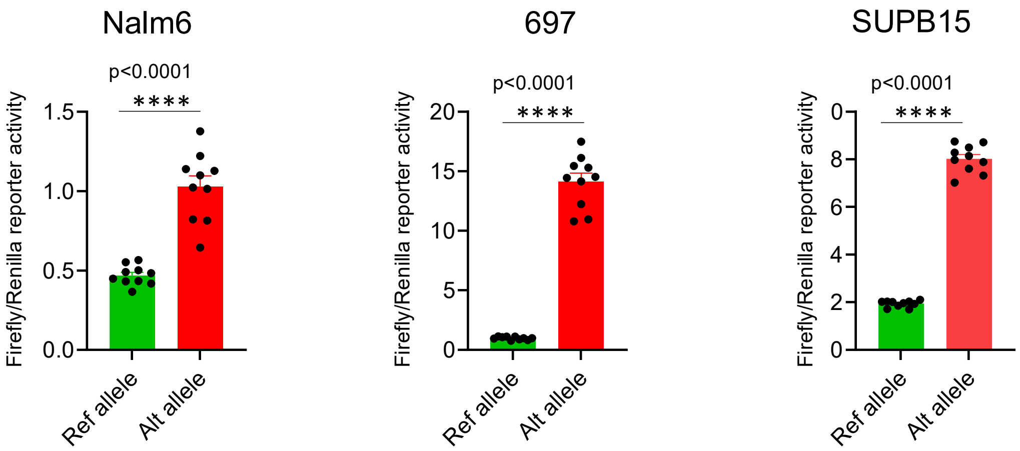

Figure S11: Luciferase reporter assay analysis of rs17481869, related to Figure 7. Firefly luciferase reporter plasmid activity for SNP rs17481869 comparing reference allele and alternative allele in Nalm6 (left), 697 (center) and SUBP15 (right) B-ALL cell lines. Firefly luciferase signal is normalized to renilla luciferase control.

Name	Sequence (5’ to 3’)
	***SLC2A9* deletion in Nalm6**
CAGE2667.SLC2A9.g7 spacer	UACAUGAGCGCACCCACGCA
CAGE2668.SLC2A9.g1 spacer	GAGACUCCUCAGCACGUGAA
CAGE2667.SLC2A9.F	CTACACGACGCTCTTCCGATCTtcaccatctcccaaggcaaaccctg
CAGE2668.SLC2A9.R	CAGACGTGTGCTCTTCCGATCTtgacctaaccgtgaccatgggcaagt
	***CDK14* deletion in SUPB15**
CAGE2657.CDK14.g9 spacer	UUUUAGACCUCUUGCAUGGA
CAGE2658.CDK14.g6 spacer	UUAUAUUGCUGGGUUGUGAU
CAGE2657.CDK14.F	CTACACGACGCTCTTCCGATCTtactttctgtactgttttattttcactgt
CAGE2658.CDK14.R	CAGACGTGTGCTCTTCCGATCTgaatctatggtccagtcctgagaaa
	***SH3BP5L* deletion in 697**
CAGE2665.SH3BP5L.g2 spacer	AUCCAUAUGUUAUUAAGGGU
CAGE2666.SH3BP5L.g5 spacer	UAAAGACAGAUUGAGAUUCC
CAGE2665.SH3BP5L.F	CTACACGACGCTCTTCCGATCTaaccctcagacactccatgaggccc
CAGE2666.SH3BP5L.R	CAGACGTGTGCTCTTCCGATCTccaggaggctcaagagaagacgtca

Table S10: List of nucleic acid sequences used for CRISPR-Cas9 deletions, related to STAR Methods.

Name	Sequence (5' to 3')
***LNX1* CRE CRISPRi silencing in Nalm6 cells**	
F_RRG421_LNX1_enh	CACCGAATAATGCACCACAGTGAGG
R_RRG421_LNX1_enh	AAACCCTCACTGTGGTGCATTATTC
F_RRG422_LNX1_enh	CACCGAGCATTCATCTGGTAAACCG
R_RRG422_LNX1_enh	AAACCGGTTTACCAGATGAATGCTC
LNX1 enh qPCR primer F	CAGTCGCTTGAAGAGGTGTG
LNX1 enh qPCR primer R	GAGGGTGCAGTAGGTGTGTC
***MAP7* CRE silencing in SEM cells**	
F_RRG423_MAP7_enh	CACCGAAAATGCTAGTTATTCACCA
R_RRG423_MAP7_enh	AAACTGGTGAATAACTAGCATTTTC
F_RRG424_MAP7_enh	CACCGTTTGCTAGCATAGAACCCAC
R_RRG424_MAP7_enh	AAACGTGGGTTCTATGCTAGCAAAC
MAP7 enh qPCR primer F	GCAGTGCGAAGCGAAACAG
MAP7 enh qPCR primer R	CAGCTTCGTGGCGTTCTTTG
***SENP6* CRE silencing in SEM cells**	
F_RRG427_SENP6_enh	CACCGAATAAAGTACATCCACCCCA
R_RRG427_SENP6_enh	AAACTGGGGTGGATGTACTTTATTC
F_RRG428_SENP6_enh	CACCGTGAAAACCAGACAAGCTGAA
R_RRG428_SENP6_enh	AAACTTCAGCTTGTCTGGTTTTCAC
SENP6 enh qPCR primer F	AACAGCAGCCCAAAGCAGTC
SENP6 enh qPCR primer R	AGGCTCCACTTGTGATTCGG

Table S11: List of sgRNA and qPCR primer sequences used for CRISPRi, related to STAR Methods.

Name	Coordinates (hg19)	Sequence
**rs17481869 Ref allele**	chr2:146124304-146124604	CTGCAATGAGTCAAGTGATTGATTTCATAAACTAAATTAG AAACTTTTAAATTTTATTTTATTTTGGCAAAAGCTGGCCTT TTATAAAATGTAAGCATCTGGGCTAGAAACTTTTTGATTT CTTGAATTTATGATTTTCTTTTATGCTTC**C**CTAGGCCTTA AGTGAAGAAAACAGGAAAGAGGCCTCCCAGAGGTGGAA GTTGTATTGTAAAAAGTGGTCTTATGTTTTAAGATAACTT GTGGGAATGATAGGAAGGATGTGAATAGGCATCCCTTG TTTTTGTGAGTTCGTTTTTTCAGA
**rs17481869 Alt allele**	chr2:146124304-146124604	CTGCAATGAGTCAAGTGATTGATTTCATAAACTAAATTAG AAACTTTTAAATTTTATTTTATTTTGGCAAAAGCTGGCCTT TTATAAAATGTAAGCATCTGGGCTAGAAACTTTTTGATTT CTTGAATTTATGATTTTCTTTTATGCTTC**A**CTAGGCCTTA AGTGAAGAAAACAGGAAAGAGGCCTCCCAGAGGTGGAA GTTGTATTGTAAAAAGTGGTCTTATGTTTTAAGATAACTT GTGGGAATGATAGGAAGGATGTGAATAGGCATCCCTTG TTTTTGTGAGTTCGTTTTTTCAGA

Table S12: Tested luciferase reporter assay DNA sequences centered on rs17481869, related to STAR Methods.
