# Supplementary material for: Epigenomic mapping reveals distinct B cell acute lymphoblastic leukemia chromatin architectures and regulators
Source: Cell Genom. 2023 Nov 20;3(12):100442. doi: 10.1016/j.xgen.2023.100442 (PMC10726428; doi:10.1016/j.xgen.2023.100442)
Supplement: Data S1. Differential TF footprint scores at subtype-enriched and subtype-depleted DAS, related to Figure 5 [file mmc2.pdf]

SUPPLEMENTAL DATA 1: Differential TF footprint scores at subtype-enriched and subtype-depleted DAS, related to Figure 5. Differential footprint score between subtype and non-subtype samples. Differential footprint score between merged B-ALL subtype and merged B-ALL non-subtype patient samples is provided on the x-axis and TF footprint significance is provided on the y-axis. Higher differential footprint scores indicate higher binding in the merged subtype group compared to all other merged non-subtype samples. TPM transcript abundance of associated TF transcript for merged subtype groups are shown as both color and size of points. Data is shown for enriched (top) and depleted (bottom) DAS specific to each subtype.

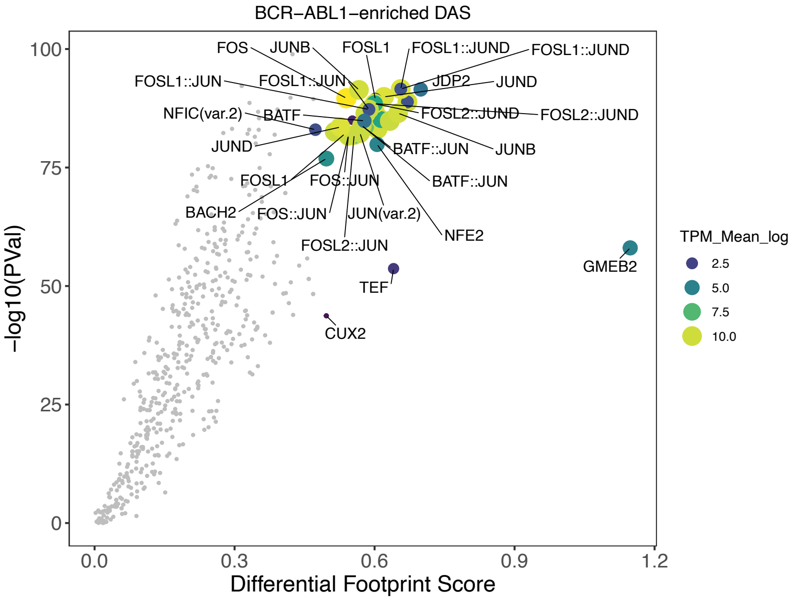

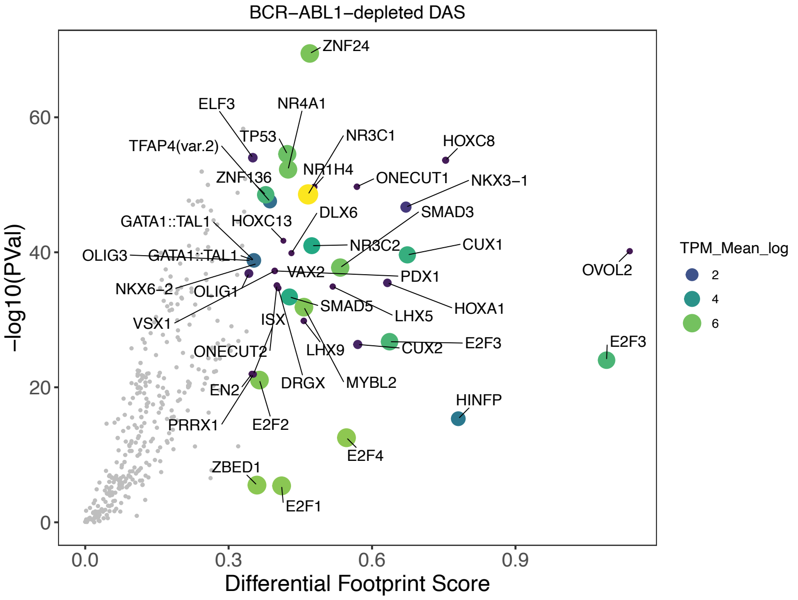

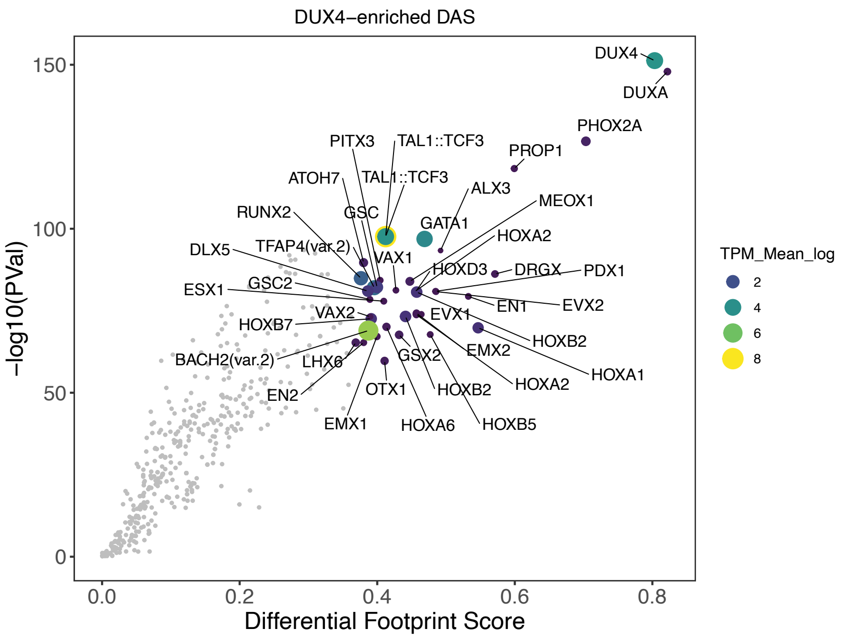
DUX4−enriched DAS
−log10(PVal)
Differential Footprint Score
0
50
100
150
0.0
0.2
0.4
0.6
0.8
DUX4
DUXA
PHOX2A
PROP1
PITX3
TAL1::TCF3
TAL1::TCF3
ATOH7
ALX3
MEOX1
RUNX2
GSC
GATA1
HOXA2
DLX5
TFAP4(var.2)
VAX1
HOXD3
DRGX
PDX1
GSC2
ESX1
EN1
EVX2
VAX2
EVX1
HOXB7
HOXB2
EMX2
BACH2(var.2)
LHX6
GSX2
HOXA1
HOXA2
EN2
OTX1
HOXB2
EMX1
HOXA6
HOXB5
TPM_Mean_log
2
4
6
8

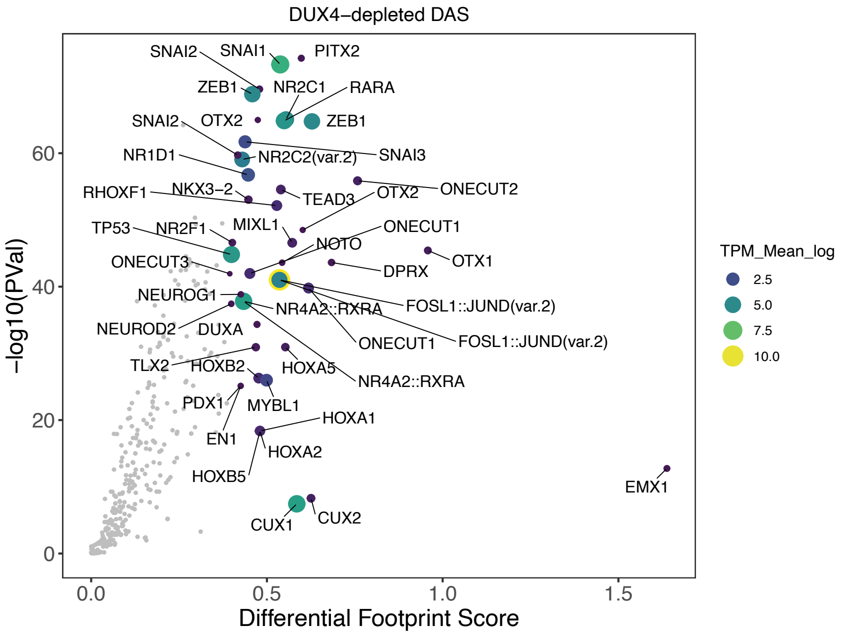
DUX4−depleted DAS
−log10(PVal)
Differential Footprint Score
0
20
40
60
0.0
0.5
1.0
1.5
SNAI2
SNAI1
PITX2
ZEB1
NR2C1
RARA
SNAI2
OTX2
ZEB1
NR1D1
SNAI3
NR2C2(var.2)
RHOXF1
NKX3−2
TEAD3
OTX2
ONECUT2
TP53
NR2F1
MIXL1
ONECUT1
NOTO
OTX1
ONECUT3
DPRX
NEUROG1
NR4A2::RXRA
FOSL1::JUND(var.2)
NEUROD2
DUXA
ONECUT1
FOSL1::JUND(var.2)
TLX2
HOXB2
HOXA5
NR4A2::RXRA
PDX1
MYBL1
HOXA1
EN1
HOXA2
HOXB5
EMX1
CUX1
CUX2
TPM_Mean_log
2.5
5.0
7.5
10.0

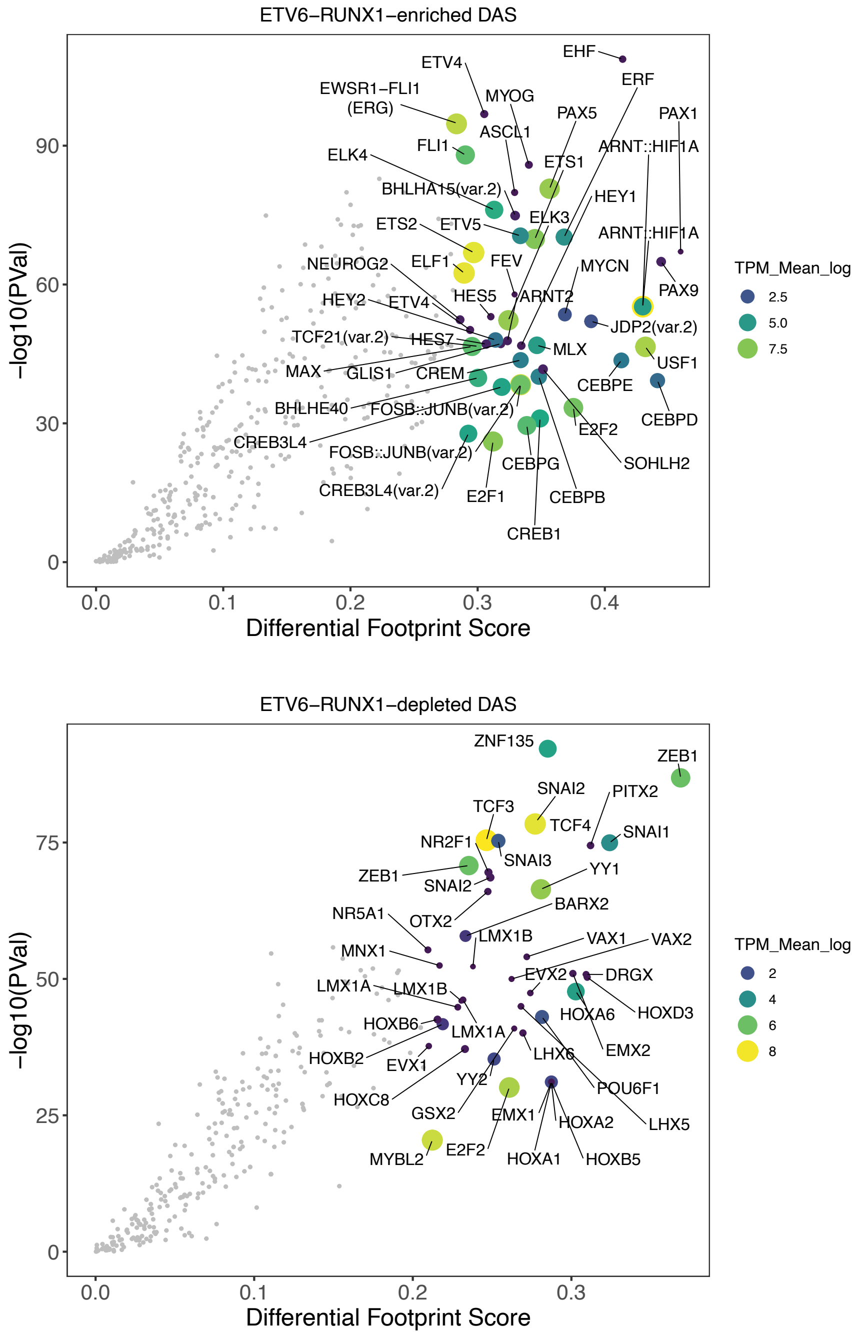
ETV6−RUNX1−enriched DAS
−log10(PVal)
Differential Footprint Score
0
30
60
90
0.0
0.1
0.2
0.3
0.4
TPM_Mean_log
2.5
5.0
7.5
EHF
ERF
ETV4
EWSR1−FLI1
(ERG)
MYOG
PAX5
PAX1
ASCL1
FLI1
ARNT::HIF1A
ELK4
ETS1
BHLHA15(var.2)
HEY1
ETS2
ETV5
ELK3
ARNT::HIF1A
ELF1
FEV
MYCN
NEUROG2
PAX9
HES5
ARNT2
HEY2
ETV4
JDP2(var.2)
TCF21(var.2)
HES7
MLX
USF1
MAX
GLIS1
CREM
CEBPE
BHLHE40
FOSB::JUNB(var.2)
CEBPD
E2F2
CREB3L4
FOSB::JUNB(var.2)
SOHLH2
CEBPG
CREB3L4(var.2)
E2F1
CEBPB
CREB1
ETV6−RUNX1−depleted DAS
−log10(PVal)
Differential Footprint Score
0
25
50
75
0.0
0.1
0.2
0.3
TPM_Mean_log
2
4
6
8
ZNF135
ZEB1
SNAI2
PITX2
TCF3
TCF4
SNAI1
NR2F1
SNAI3
ZEB1
YY1
SNAI2
BARX2
NR5A1
OTX2
LMX1B
VAX1
VAX2
MNX1
EVX2
DRGX
LMX1A
LMX1B
HOXA6
HOXD3
HOXB6
LMX1A
HOXB2
EVX1
LHX6
EMX2
YY2
POU6F1
HOXC8
GSX2
EMX1
HOXA2
LHX5
E2F2
MYBL2
HOXA1
HOXB5

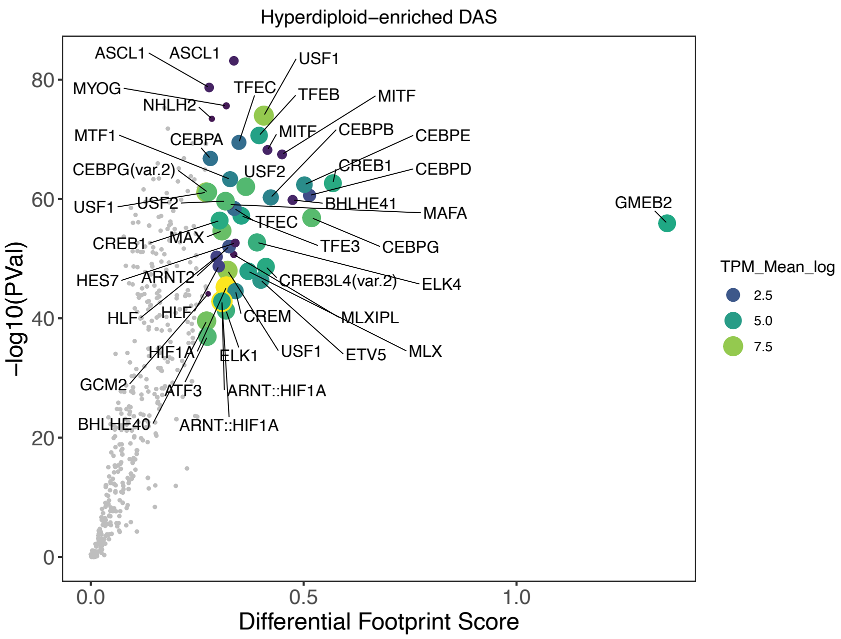
Hyperdiploid−enriched DAS
ASCL1
ASCL1
USF1
MYOG
TFEC
TFEB
MITF
NHLH2
MITF
CEBPB
CEBPE
MTF1
CEBPA
CREB1
CEBPD
CEBPG(var.2)
USF2
BHLHE41
GMEB2
USF1
USF2
MAFA
TFEC
CREB1
MAX
TFE3
CEBPG
HES7
ARNT2
CREB3L4(var.2)
ELK4
HLF
HLF
CREM
MLXIPL
HIF1A
ELK1
USF1
ETV5
MLX
GCM2
ATF3
ARNT::HIF1A
BHLHE40
ARNT::HIF1A
−log10(PVal)
Differential Footprint Score
0
20
40
60
80
0.0
0.5
1.0
TPM_Mean_log
2.5
5.0
7.5

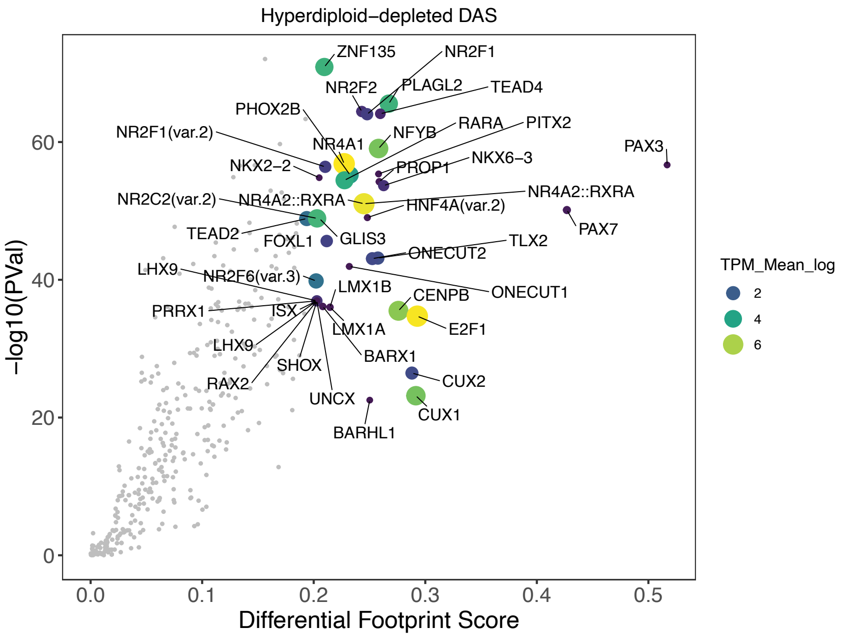
Hyperdiploid−depleted DAS
ZNF135
NR2F1
NR2F2
PLAGL2
TEAD4
PHOX2B
RARA
PITX2
NR2F1(var.2)
NR4A1
NFYB
PAX3
NKX2−2
PROP1
NKX6−3
NR2C2(var.2)
NR4A2::RXRA
NR4A2::RXRA
HNF4A(var.2)
PAX7
TEAD2
FOXL1
GLIS3
ONECUT2
TLX2
LHX9
NR2F6(var.3)
ONECUT1
LMX1B
CENPB
PRRX1
ISX
LMX1A
E2F1
LHX9
BARX1
SHOX
RAX2
CUX2
UNCX
CUX1
BARHL1
−log10(PVal)
Differential Footprint Score
0
20
40
60
0.0
0.1
0.2
0.3
0.4
0.5
TPM_Mean_log
2
4
6

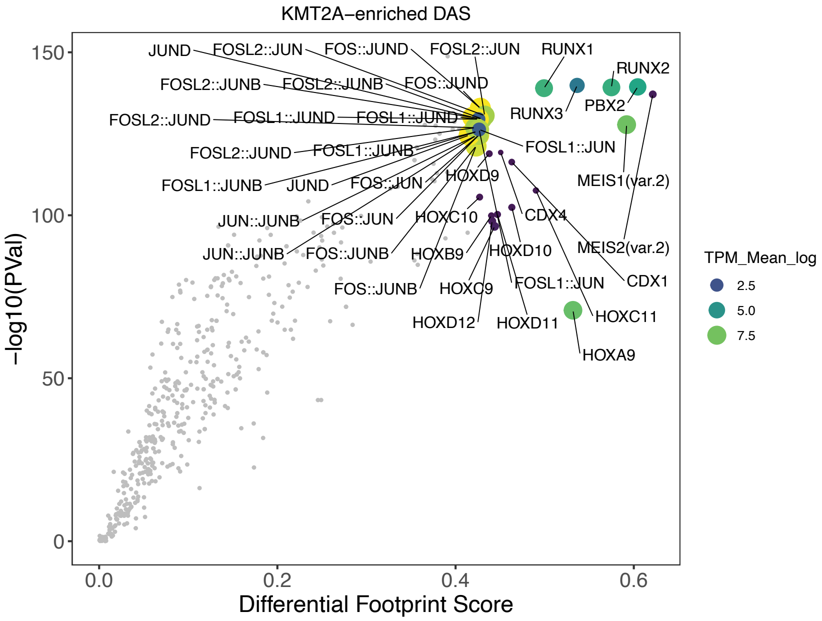
KMT2A−enriched DAS
−log10(PVal)
Differential Footprint Score
TPM_Mean_log
2.5
5.0
7.5
JUND
FOSL2::JUN
FOS::JUND
FOSL2::JUN
RUNX1
RUNX2
FOSL2::JUNB
FOSL2::JUNB
FOS::JUND
RUNX3
PBX2
FOSL2::JUND
FOSL1::JUND
FOSL1::JUND
FOSL1::JUN
FOSL2::JUND
FOSL1::JUNB
MEIS1(var.2)
FOSL1::JUNB
JUND
FOS::JUN
HOXD9
JUN::JUNB
FOS::JUN
HOXC10
CDX4
JUN::JUNB
FOS::JUNB
HOXB9
HOXD10
MEIS2(var.2)
FOS::JUNB
HOXC9
FOSL1::JUN
CDX1
HOXD12
HOXD11
HOXC11
HOXA9

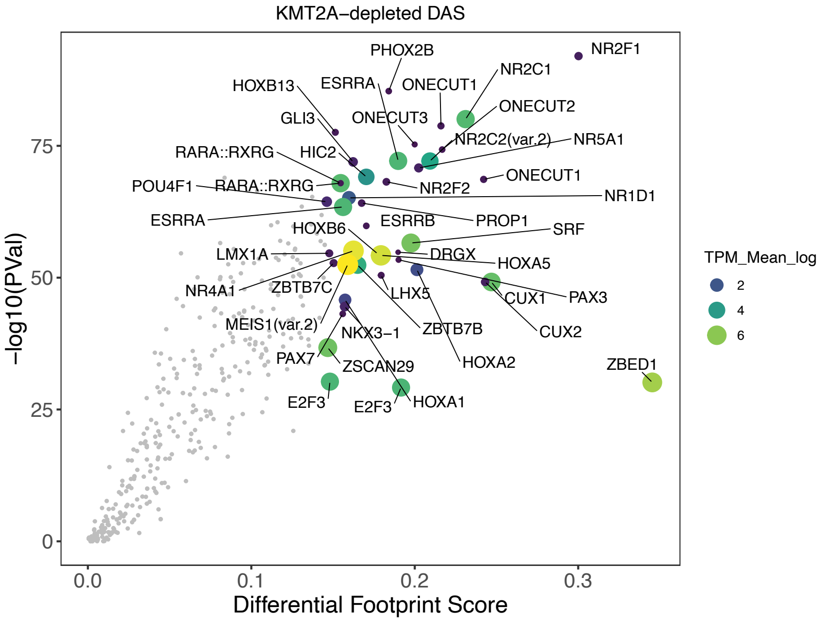
KMT2A−depleted DAS
−log10(PVal)
Differential Footprint Score
TPM_Mean_log
2
4
6
PHOX2B
NR2F1
NR2C1
HOXB13
ESRRA
ONECUT1
ONECUT2
GLI3
ONECUT3
NR2C2(var.2)
NR5A1
RARA::RXRG
HIC2
ONECUT1
POU4F1
RARA::RXRG
NR2F2
NR1D1
ESRRA
PROP1
HOXB6
ESRRB
SRF
LMX1A
DRGX
HOXA5
NR4A1
ZBTB7C
LHX5
CUX1
PAX3
MEIS1(var.2)
NKX3−1
ZBTB7B
CUX2
PAX7
ZSCAN29
HOXA2
ZBED1
E2F3
E2F3
HOXA1

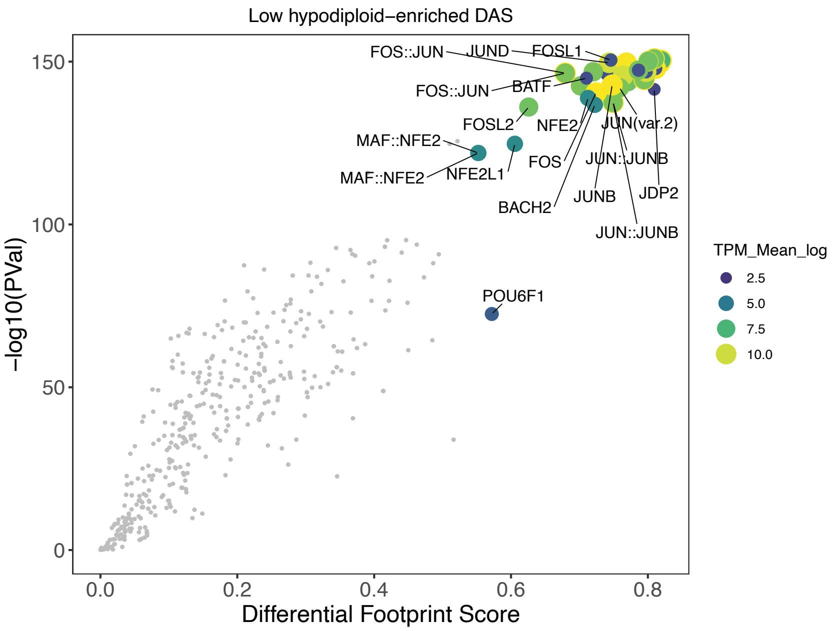
Low hypodiploid–enriched DAS
−log10(PVal)
Differential Footprint Score
TPM_Mean_log
2.5
5.0
7.5
10.0
FOS::JUN
JUND
FOSL1
FOS::JUN
BATF
FOSL2
NFE2
JUN(var.2)
MAF::NFE2
MAF::NFE2
NFE2L1
FOS
JUN::JUNB
JUNB
JDP2
BACH2
JUN::JUNB
POU6F1

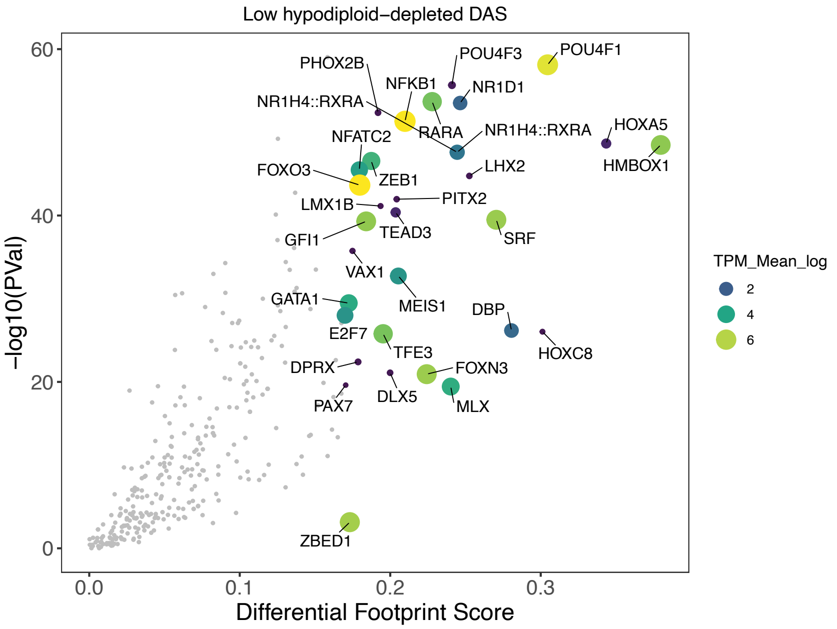
Low hypodiploid–depleted DAS
−log10(PVal)
Differential Footprint Score
TPM_Mean_log
2
4
6
PHOX2B
POU4F3
POU4F1
NFKB1
NR1D1
NR1H4::RXRA
RARA
NR1H4::RXRA
HOXA5
NFATC2
HMBOX1
FOXO3
ZEB1
LHX2
LMX1B
PITX2
TEAD3
GFI1
SRF
VAX1
GATA1
MEIS1
DBP
E2F7
HOXC8
TFE3
DPRX
FOXN3
DLX5
PAX7
MLX
ZBED1

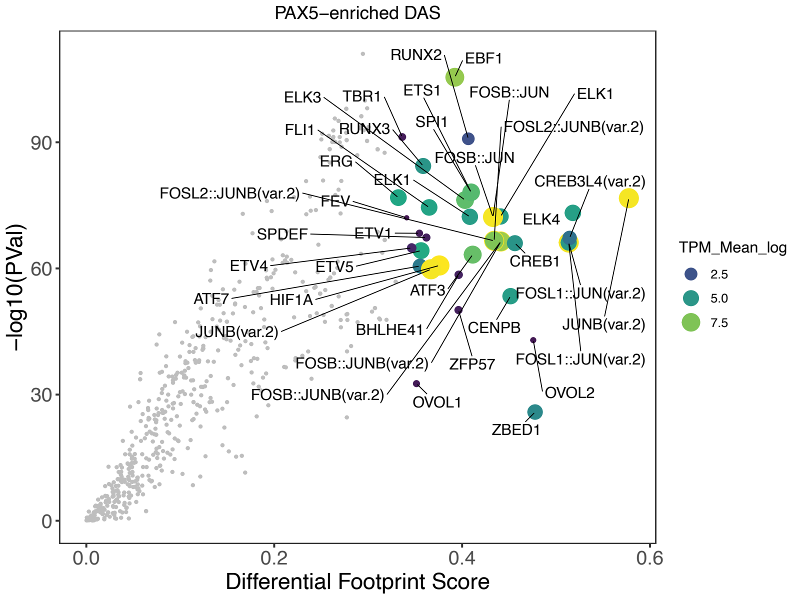

PAX5−enriched DAS
RUNX2
EBF1
ELK3
TBR1
ETS1
FOSB::JUN
ELK1
FLI1
RUNX3
SPI1
FOSL2::JUNB(var.2)
ERG
FOSB::JUN
CREB3L4(var.2)
ELK1
FOSL2::JUNB(var.2)
FEV
ELK4
SPDEF
ETV1
CREB1
ETV4
ETV5
ATF3
FOSL1::JUN(var.2)
ATF7
HIF1A
CENPB
JUNB(var.2)
BHLHE41
JUNB(var.2)
FOSB::JUNB(var.2)
ZFP57
FOSL1::JUN(var.2)
FOSB::JUNB(var.2)
OVOL1
OVOL2
ZBED1
−log10(PVal)
Differential Footprint Score
0
30
60
90
0.0
0.2
0.4
0.6
TPM_Mean_log
2.5
5.0
7.5

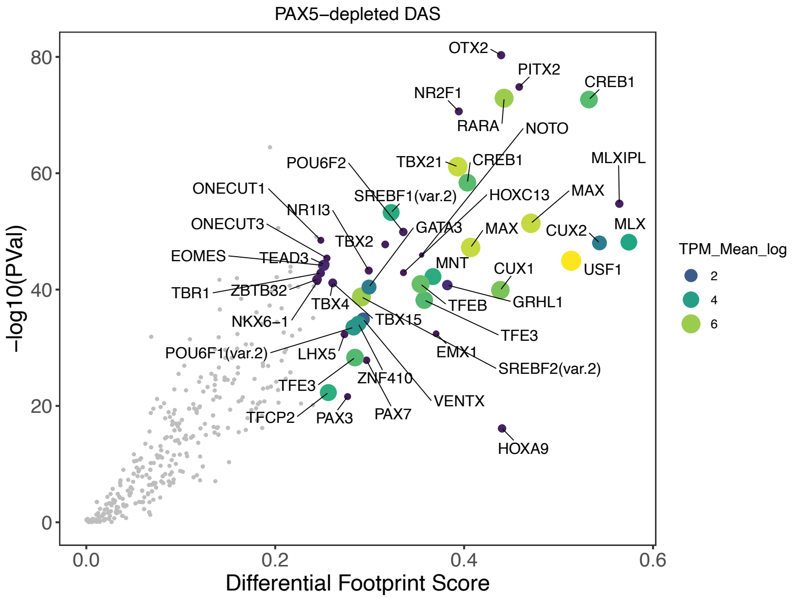

PAX5−depleted DAS
OTX2
PITX2
CREB1
NR2F1
RARA
NOTO
POU6F2
TBX21
CREB1
MLXIPL
ONECUT1
SREBF1(var.2)
HOXC13
MAX
NR1I3
ONECUT3
GATA3
MAX
CUX2
MLX
TBX2
EOMES
TEAD3
MNT
CUX1
USF1
TBR1
ZBTB32
TBX4
TFEB
GRHL1
NKX6−1
TBX15
TFE3
POU6F1(var.2)
LHX5
EMX1
SREBF2(var.2)
ZNF410
TFE3
VENTX
TFCP2
PAX3
PAX7
HOXA9
−log10(PVal)
Differential Footprint Score
0
20
40
60
80
0.0
0.2
0.4
0.6
TPM_Mean_log
2
4
6

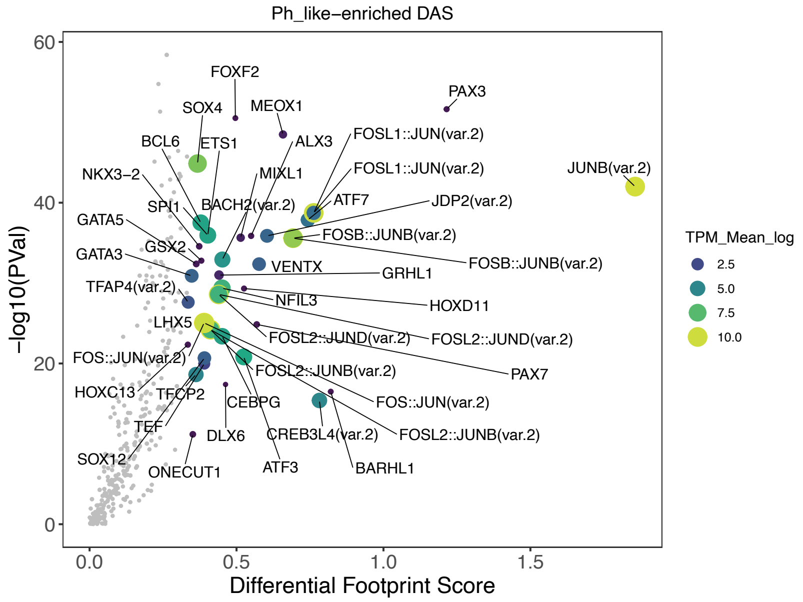
Ph_like−enriched DAS
−log10(PVal)
Differential Footprint Score
TPM_Mean_log
2.5
5.0
7.5
10.0
FOXF2
PAX3
SOX4
MEOX1
BCL6
ETS1
ALX3
FOSL1::JUN(var.2)
FOSL1::JUN(var.2)
JUNB(var.2)
NKX3−2
MIXL1
SPI1
BACH2(var.2)
ATF7
JDP2(var.2)
GATA5
GSX2
FOSB::JUNB(var.2)
FOSB::JUNB(var.2)
GATA3
VENTX
GRHL1
TFAP4(var.2)
NFIL3
HOXD11
LHX5
FOSL2::JUND(var.2)
FOSL2::JUND(var.2)
FOS::JUN(var.2)
PAX7
FOSL2::JUNB(var.2)
HOXC13
TFCP2
CEBPG
FOS::JUN(var.2)
TEF
DLX6
CREB3L4(var.2)
FOSL2::JUNB(var.2)
SOX12
ONECUT1
ATF3
BARHL1

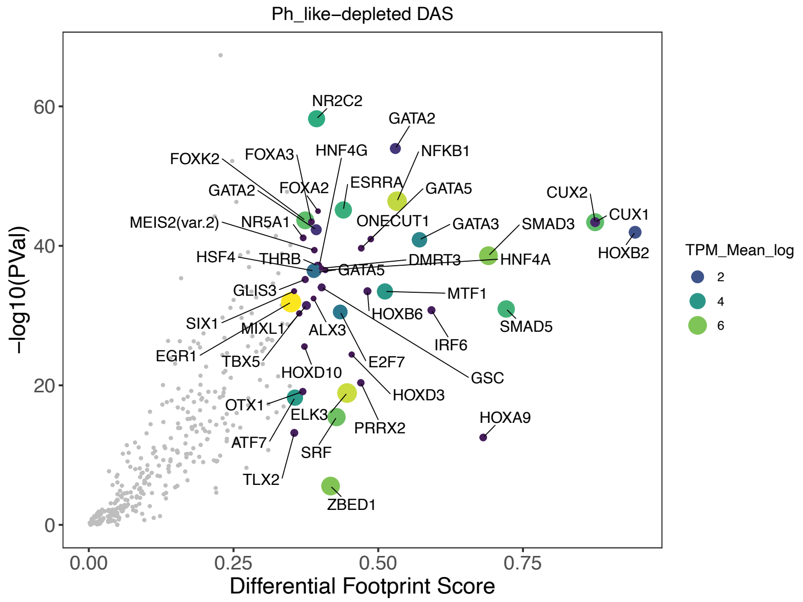
Ph_like−depleted DAS
−log10(PVal)
Differential Footprint Score
TPM_Mean_log
2
4
6
NR2C2
GATA2
FOXK2
FOXA3
HNF4G
NFKB1
GATA2
FOXA2
ESRRA
GATA5
CUX2
CUX1
MEIS2(var.2)
NR5A1
ONECUT1
GATA3
SMAD3
HOXB2
HSF4
THRB
GATA5
DMRT3
HNF4A
GLIS3
MTF1
HOXB6
SIX1
MIXL1
ALX3
IRF6
SMAD5
EGR1
TBX5
E2F7
HOXD10
GSC
HOXD3
OTX1
ELK3
PRRX2
HOXA9
ATF7
SRF
TLX2
ZBED1

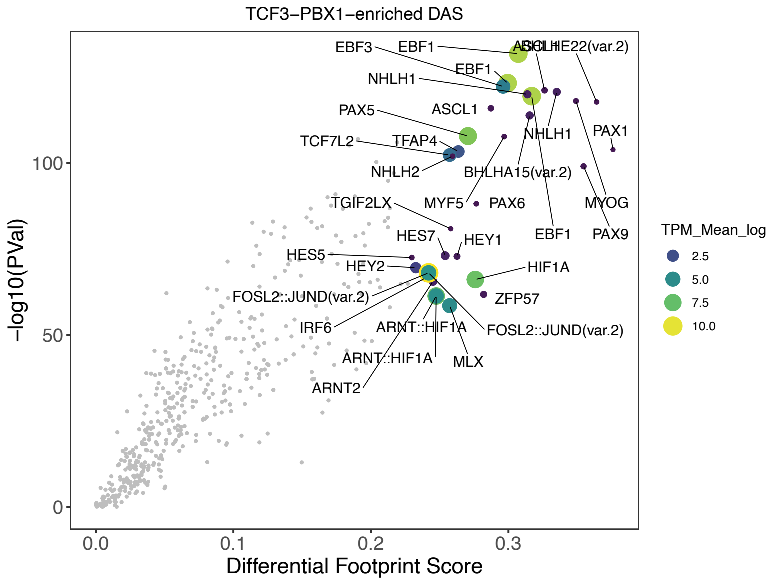
TCF3−PBX1−enriched DAS
−log10(PVal)
Differential Footprint Score
TPM_Mean_log
2.5
5.0
7.5
10.0
0
50
100
0.0
0.1
0.2
0.3
EBF3
EBF1
NHLH1
PAX5
TCF7L2
TFAP4
NHLH2
TGIF2LX
HES5
HEY2
FOSL2::JUND(var.2)
IRF6
ARNT2
ARNT::HIF1A
MLX
ZFP57
HIF1A
HEY1
HES7
MYF5
PAX6
BHLHA15(var.2)
NHLH1
ASCL1
EBF1
PAX1
MYOG
PAX9

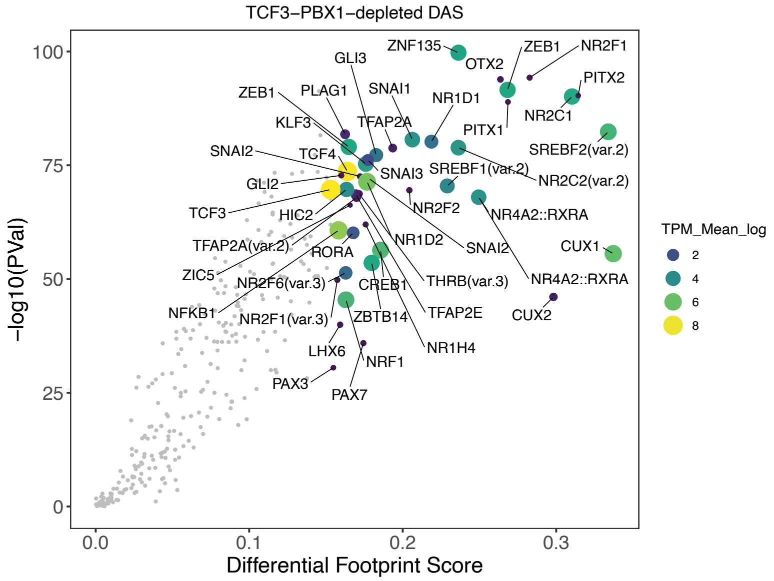
TCF3−PBX1−depleted DAS
−log10(PVal)
Differential Footprint Score
TPM_Mean_log
2
4
6
8
0
25
50
75
100
0.0
0.1
0.2
0.3
ZNF135
ZEB1
NR2F1
GLI3
OTX2
PITX2
ZEB1
PLAG1
SNAI1
NR1D1
KLF3
TFAP2A
PITX1
NR2C1
SREBF2(var.2)
SNAI2
TCF4
SNAI3
SREBF1(var.2)
NR2C2(var.2)
GLI2
NR2F2
TCF3
HIC2
NR4A2::RXRA
TFAP2A(var.2)
NR1D2
SNAI2
CUX1
RORA
ZIC5
NR2F6(var.3)
CREB1
THRB(var.3)
NR4A2::RXRA
NFKB1
NR2F1(var.3)
ZBTB14
TFAP2E
CUX2
LHX6
NR1H4
NRF1
PAX3
PAX7

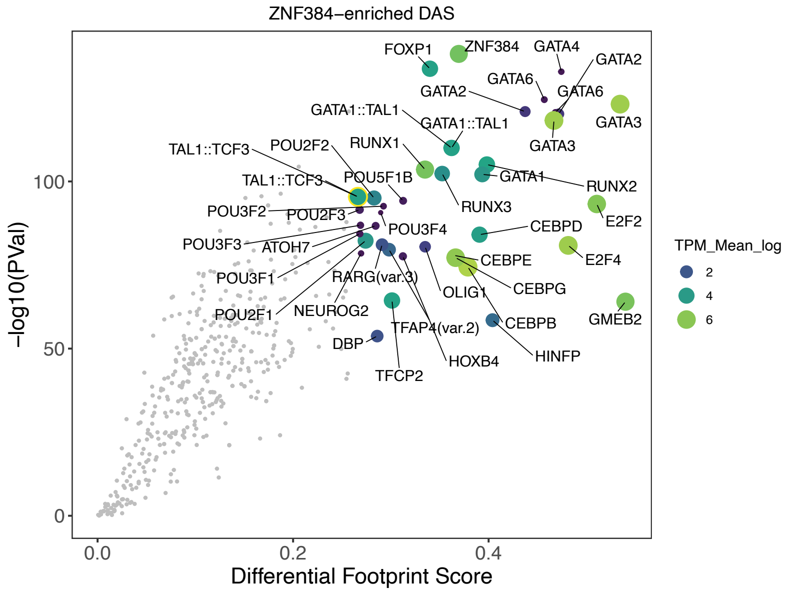
ZNF384−enriched DAS
−log10(PVal)
Differential Footprint Score
0.0
0.2
0.4
0
50
100
FOXP1
ZNF384
GATA4
GATA2
GATA6
GATA2
GATA6
GATA1::TAL1
GATA1::TAL1
GATA3
GATA3
TAL1::TCF3
POU2F2
RUNX1
TAL1::TCF3
POU5F1B
GATA1
RUNX2
POU3F2
POU2F3
RUNX3
E2F2
POU3F4
CEBPD
POU3F3
ATOH7
CEBPE
E2F4
POU3F1
RARG(var.3)
OLIG1
CEBPG
POU2F1
NEUROG2
CEBPB
GMEB2
TFAP4(var.2)
DBP
HINFP
HOXB4
TFCP2
TPM_Mean_log
2
4
6

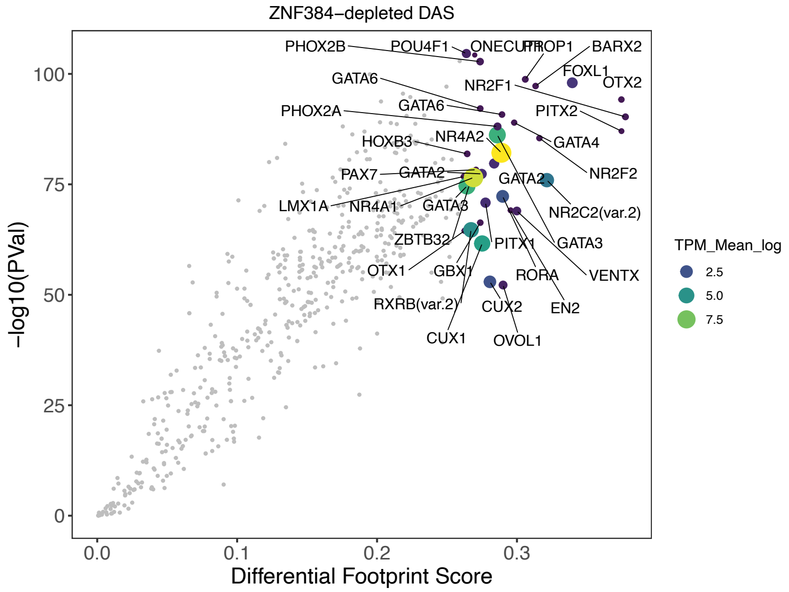
ZNF384−depleted DAS
−log10(PVal)
Differential Footprint Score
0.0
0.1
0.2
0.3
0
25
50
75
100
PHOX2B
POU4F1
BARX2
FOXL1
OTX2
GATA6
NR2F1
PHOX2A
GATA6
PITX2
HOXB3
NR4A2
GATA4
PAX7
GATA2
NR2F2
GATA2
LMX1A
NR4A1
GATA3
NR2C2(var.2)
ZBTB32
PITX1
GATA3
OTX1
GBX1
RORA
VENTX
RXRB(var.2)
CUX2
EN2
CUX1
OVOL1
TPM_Mean_log
2.5
5.0
7.5
